# Polyclonality and metabolic heterogeneity in a colorectal tumor model

**DOI:** 10.1016/j.isci.2025.113090

**Published:** 2025-07-10

**Authors:** Pierre Delamotte, Mickael Poidevin, Yan Jaszczyszyn, Arnaud Le Rouzic, Jacques Montagne

**Affiliations:** 1 Institut for Integrative Biology of the Cell (I2BC), UMR 9198, Université Paris-Saclay, CNRS, CEA, 91190 Gif-sur-Yvette, France; 2 Biologie Fonctionnelle et Adaptative (BFA), UMR 8251, Université Paris-Cité, CNRS, 75113 Paris, France; 3 Université Paris-Saclay, CNRS, IRD, UMR EGCE, 91190 Gif-sur-Yvette, France; 4 Institut de Génomique Fonctionnelle de Lyon, ENS de Lyon, CNRS UMR5242, UCBL1, 69007 Lyon, France

**Keywords:** Cancer, Experimental models in systems biology, Metabolic flux analysis

## Abstract

The monoclonal origin of cancer is widely accepted, although numerous studies suggest that some are of polyclonal origin. Loss of checkpoints in transformed cells gives rise to carcinomas comprising a wide diversity of cell types that fulfill distinct, even complementary, metabolic functions, contrasting with a hypothetical monoclonal origin. Here, using a *Drosophila* intestinal tumor model, we show that, despite an identical genetic background, these tumors (1) comprise a conserved set of different metabolic-specialized clusters; (2) are always polyclonal and derive from several clones characterized by distinct metabolic specificity; (3) depend on motility of the founder clones for their growth; and (4) share metabolic needs similar to those of human cancers. In summary, our study indicates that, in this model, tumor formation always requires assembly between founder clones potentially providing distinct cellular functions, as visualized by their metabolic heterogeneity. Thus, this polyclonal assembly would constitute a critical step of tumor progression.

## Highlights

• Polyclonality is the exclusive mode of formation of these intestinal tumors
• These tumors organize into a conserved set of metabolically distinct sub-clusters
• Metabolic heterogeneity is observed between tumor founder clones
• Genes regulating cell mobility are required for the growth of these tumors

## Graphical abstract

**Figure:**
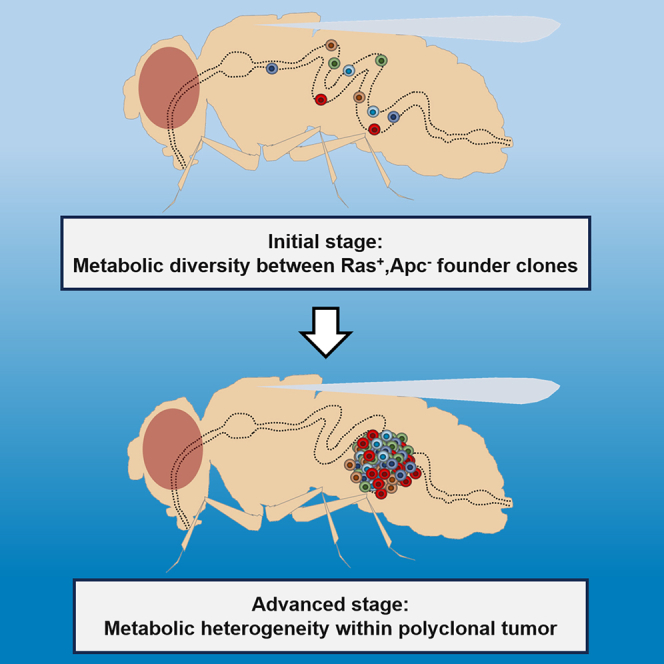

## Introduction

The commonly accepted cancer paradigm postulates that cancer originates from a single immortalized cell, which, through checkpoint-defective divisions, results in a tumor containing a great diversity of cell types.^1^^,^^2^^,^^3^ Conversely, other studies suggest that some tumors are polyclonal,^4^ generating a great debate about the clonal origin of cancers.^5^^,^^6^^,^^7^^,^^8^ Our present study, initially designed to characterize the metabolic requirements of a *Drosophila* intestinal tumor model, prompted us to investigate its potential polyclonal origin.

Carcinomas, which are tumors of epithelial origin, represent more than 80% of cancer cases in humans. Apart from inherited familial syndromes, carcinomas arise from sporadic transformations and progress through changes in cellular properties.^9^^,^^10^^,^^11^ The progressive oncogenic transformation is associated with loss of checkpoints favoring defects in DNA repair and cell division, thereby increasing mutation frequency and aneuploidy occurrence in a feedforward manner.^12^^,^^13^ The great diversity of transformed cells within a tumor contrasts with its monoclonal origin and constitutes a critical challenge for cancer therapy since some cell types potentially resistant to drug treatment can be selected, resulting later in subsequent cancer relapse.^1^^,^^14^ This clonal paradox also contrasts with studies reporting that metastases are more likely to survive and proliferate when comprising several sub-clones from the primary tumor.^15^^,^^16^^,^^17^^,^^18^^,^^19^ Conversely, experimental intestinal cancer models in mice have revealed the occurrence of polyclonal tumors.^20^^,^^21^^,^^22^^,^^23^ Additionally, several studies argue that the monoclonal origin dogma must be reconsidered in several cancer types.^4^^,^^24^^,^^25^^,^^26^^,^^27^^,^^28^^,^^29^ Given that most cancers comprise distinct cell types fulfilling complementary cellular functions to favor tumor progression, determining the monoclonal or polyclonal origin of each cancer type is crucial for establishing efficient drug treatments.

Compared to healthy cells, cancer cells exhibit metabolic changes. Pioneer studies of Warburg^30^ and Crabtree^31^ reported the anaerobic glycolytic consumption of glucose to lactate by cancer cells and glycolysis-induced repression of respiratory flux in tumors. However, tumor growth also relies on electron transfer complexes (ETCs) of the mitochondrial respiratory chains,^32^^,^^33^ while mutations impairing mitochondrial functions have been reported in some cancers.^34^ Numerous recent studies have reported that tumor cells tend to increase several metabolic pathways, including (1) alternative glycolytic pathways; (2) the pentose phosphate pathway (PPP); (3) glutamine consumption to feed tricarboxylic acid cycle (TCA) anaplerosis; (4) fatty acid (FA) synthesis; and (5) glycogen synthesis. Such metabolic changes in tumor cells provide interesting targets for drug treatment, but these changes appear specific to tumor cell type. Therefore, despite many studies, metabolic changes in cancer cells still need deeper investigations.

In humans, colorectal cancers (CRCs) are often associated with loss of the tumor suppressors p53 and Apc (adenomatous polyposis coli) and with oncogenic transformation of the proto-oncogene Ras.^26^^,^^35^ Likely, because of a high regeneration rate of intestinal cells, several CRCs have been reported being of polyclonal origin.^26^^,^^36^ Based on genetic alterations of human CRC, a *Drosophila* model has been developed, where somatically recombined intestinal stem cells (ISCs) are mutated for the two *Drosophila apc* homologues and express the RasV12 oncogene.^37^ These recombined cells can form intestinal tumors that also express GFP, providing an accurate monitoring of tumor growth.^38^^,^^39^ The *Drosophila* digestive tract comprises the foregut, the midgut where ISCs reside, and the hindgut. Furthermore, these tumors mostly develop in the anterior midgut,^37^^,^^38^ where, as in the human colon, the microbial titer is the highest. Here, while investigating the metabolism of these intestinal tumors, we discovered that several pathways, were required to a variable extent for tumor growth, including fermenting and respiratory metabolisms, which, based on the Crabtree effect,^31^ are not supposed to be concurrently active in the same cells. In-depth analysis, including a probabilistic approach, revealed that these tumors always comprise distinct metabolic clusters and are of polyclonal origin.

## Results

### Screening for metabolic requirements of the tumor model

**Figure 1:**
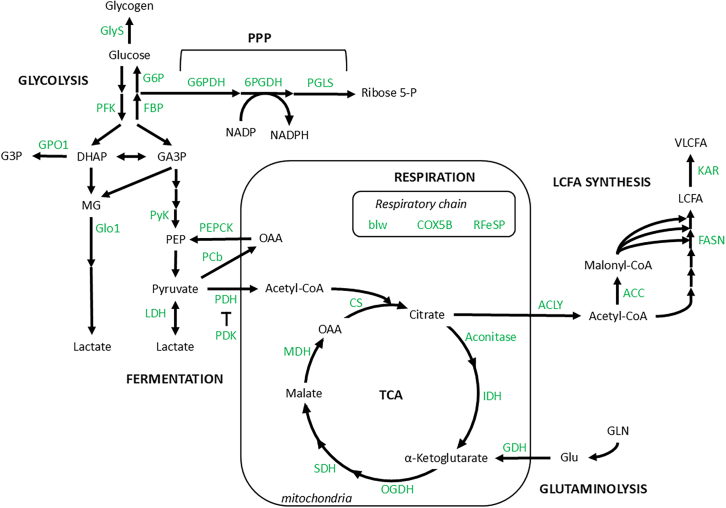
Metabolic network investigated The network shows the links between glycolysis, PPP, mitochondrial energy metabolism, FA synthesis, and gluconeogenesis. The enzymes studied are in green color. For concision, only the main pathways are represented.

To investigate the metabolic requirements, we recombined *UAS-RNAi* transgenes with *UAS-RasV12* to knockdown metabolic enzymes and regulators in tumor cells only. In this way, following heat-shock induction, recombined cells that become tumorous and GFP^+^ also express a *UAS-RNAi* transgene. Targeted enzymes were selected to challenge anaerobic glycolysis, TCA, ETCs, mitochondrial-related metabolism of amino acids, the PPP, one-carbon metabolism, FA synthesis, triacylglycerol (TAG) and glycogen storage, and gluconeogenesis (Figure 1). Tumors were heat-shock-induced on 2–3 days newly emerged adult flies and tumor growth was monitored 21 days post-induction (dpi) by flow cytometry.^38^^,^^39^ The statistical significance of differences in the proportions of tumor cells was assessed with a mixed-effect binomial generalized linear model (GLM).

#### Glucose fate via glycolysis, fermentation, and glycogen synthesis

**Figure 2:**
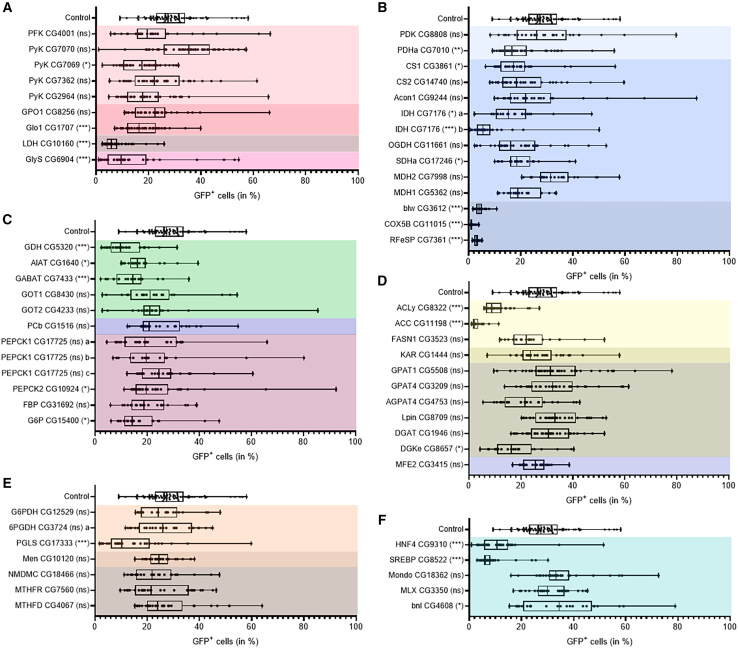
Micro-screen for metabolic genes FACS measurement of GFP^+^, tumor cells in 21-dpi midguts (A) Glucose fate: glycolysis (light red), glycolytic-associated enzymes (red), lactic fermentation (brown), and glycogen synthesis (pink). (B) Respiration: pyruvate entry into mitochondria (light blue), TCA (blue), and ETCs (dark blue). (C) Anaplerotic and cataplerotic-linked reactions: amino acid-related metabolism (green), pyruvate-support of anaplerosis (violet), and gluconeogenesis (purple). (D) Lipid metabolism: LCFA synthesis (yellow), VLCFA synthesis (dark-yellow), TAG/phospholipid synthesis (brown), and peroxisomal β-oxidation (violet). (E) NADPH producing pathways: PPP (orange), malic enzyme (dark orange), and one-carbon metabolism (brown). (F) Metabolic regulators (cyan). Controls expressing no RNAi have been processed all along the screening duration and pooled together for all series; *x* axis is the percentage of GFP^+^ cells; each box represents the distribution (25%, 50%, and 75% quartiles) of at least 100 midguts. Each dot is a replicate containing five midguts. *p* values were calculated from a generalized linear model (overdispersed binomial), the control being considered as the intercept of the model. *p* values were corrected for multiple testing by the Holm-Bonferoni method; ∗∗∗ < *p* = 0.001 < ∗∗ < *p* = 0.01 < ∗ < *p* = 0.05 < (ns).

To challenge glycolysis, we focused on PFK (phosphofructokinase) and Pyk (pyruvate kinase) that reside in the glycolytic preparatory and payoff phases, respectively. We also challenged (1) LDH (lactate dehydrogenase) that reversibly converts pyruvate to lactate; (2) GPO1 (glycerophosphate oxidase 1) that reversibly converts dihydroxyacetone phosphate (DHAP) to glycerol-3P; (3) Glo1, an enzyme of the glyoxalase complex that neutralizes the glycolytic bi-product methylglyoxal^40^; and (4) GlyS (glycogen synthase) that catalyzes glycogen synthesis. Moderate effects were observed for glycolytic enzymes (Figure 2A); PFK knockdown tended to reduce tumor growth, although not significantly, while an unconventional Pyk (CG7069), but not the conventional one (CG7070), significantly reduced tumor growth. Additionally, knockdown of Glo1, but not GPO1, significantly reduced tumor growth (Figure 2A). In contrast, GlyS or LDH knockdown provoked a strong suppression of tumor growth (Figure 2A). These results indicate that glycolysis contributes, although not critically, to the growth of these tumors, whereas the extreme growth suppression of LDH and GlyS knockdown indicates that lactate metabolism and glycogen synthesis are critical for these tumors.

#### TCA and ETCs

To challenge mitochondrial activity, we knocked down several enzymes of the TCA and three ETC components: RFeSP, COX5B, and blw, which are subunits of complexes III, IV, and V, respectively.^34^ We also challenged PDH (pyruvate dehydrogenase) that provides the acetyl-CoA substrate to TCA and its negative regulator pyruvate dehydrogenase kinase (PDK). Knockdown of PDHa, but not PDK, provoked a significant reduction of tumor growth (Figure 2B), supporting that the TCA is required. Knockdown of CS1 (citrate synthase 1), IDH (isocitrate dehydrogenase), or SDHa (succinate dehydrogenase-a) induced a significant drop in tumor growth (Figure 2B). In contrast, knockdown of Acon1 (aconitase1), OGDH (oxoglutarate dehydrogenase), or Mdh (malate dehydrogenase) did not significantly reduce tumor growth (Figure 2B), potentially because of gene duplication, as clearly established for Mdh1 and Mdh2. Nonetheless, the dramatic reduction of tumor growth due to knockdown of blw, COX5B, or RFeSP (Figure 2B) definitely confirmed the critical need for mitochondrial respiratory fluxes. However, that the knockdown of ETC components, but not TCA intermediates, almost abolished tumor growth, suggests that the drop of NADH substrate and the subsequent ATP scarcity is not as toxic as electron transfer defect for the mitochondria.

#### Transaminases, anaplerosis, and gluconeogenesis

Gluconeogenesis first bypasses the irreversible final glycolytic step via the cataplerotic reaction catalyzed by PEPCK to convert OAA (oxaloacetate) into PEP (phosphoenolpyruvate). Several glycolytic reactions are reversible except the dephosphorylation of fructose-1,6-bisphosphate and of glucose-6P by fructose 1,6-biphosphatase (FBP) and G6P, respectively. Therefore, to challenge gluconeogenesis, we knocked down PEPCK, FBP, and G6P. Gluconeogenesis is a cataplerotic process, whereas transamination and pyruvate carboxylation catalyze anaplerotic reactions to replenish TCA intermediates.^41^ To challenge TCA anaplerosis, we knocked down PCb (pyruvate carboxylase); the transaminases AIAT (alanine aminotransferase), GABAT (γ-aminobutyric acid transaminase), and GOT (glutamate oxaloacetate transaminase); and GDH (glutamate dehydrogenase). Knockdown of GDH, AIAT, or GABAT, but not GOT1, GOT2, or PCb, significantly reduced tumor growth (Figure 2C). Additionally, knockdown of PEPCK2 or G6P resulted in a significant reduction of tumor growth (Figure 2C), whereas PEPCK1 or FBP knockdown tended to reduce—although not significantly—tumor growth (Figure 2C). These results indicate that, as reported in numerous cancers,^42^ anaplerosis via glutamine consumption is required for the growth of the intestinal tumor model. This anaplerotic activity potentially compensates for the cataplerotic effect of gluconeogenesis.

#### Lipid metabolism

To challenge FA synthesis, we knocked down ACLy (ATP citrate lyase), ACC (acetyl-CoA carboxylase), and FASN (fatty acid synthase), three enzymes successively required for synthesis of long-chain FA (LCFA), and KAR (keto-acyl-reductase), a subunit of the elongase complex that catalyzes synthesis of very-long-chain FAs (VLCFAs).^40^^,^^43^^,^^44^ We also challenged (1) GPAT1, GPAT4, AGPAT4, lipin, and diacylglycerol acyltransferase (DGAT), potentially required for TAG and/or phospholipid synthesis; (2) the peroxisomal multifunctional enzyme MFE2; and (3) DGKε that catalyze phosphatidic acid (PA) production at the endoplasmic reticulum (ER) to sustain phosphatidylinositol-dependent cell signaling.^45^^,^^46^ Knockdown of ACLY or ACC strongly reduced tumor growth (Figure 2D), whereas knockdown of FASN1, one of the three *Drosophila* FASN enzymes, marginally reduced tumor growth (Figure 2D). However, KAR knockdown did not affect tumor growth (Figure 2D), suggesting that LCFA but not VLCFA synthesis is necessary for tumor growth. None of the enzymes potentially involved in TAG and/or phospholipid synthesis significantly reduced tumor growth, potentially because of gene redundancy (Figure 2D). Knockdown of MFE2 did not affect tumor growth (Figure 2D), indicating that peroxisome β-oxidation is not necessary for these tumors. Conversely, DGKε knockdown significantly reduced tumor growth (Figure 2D) indicating that phosphatidylinositol-dependent signaling is likely required for these tumors.

#### Nucleotide and NADPH synthesis

Nucleotide synthesis requires 5C sugars produced by the PPP, which concurrently produces NADPH, a critical coenzyme for FA synthesis and for neutralizing reactive oxygen species. NADPH can also be produced via the malic enzyme or one-carbon metabolism,^47^ the latter being also required for nucleotide synthesis. Therefore, we knocked down G6PDH (glucose-6P dehydrogenase), 6PGDH (6P-gluconate dehydrogenase), and PGLS (6P-gluconolactonase) to challenge the PPP; NMDMC (methylenetetrahydrofolate dehydrogenase), MTHFR (methylenetetrahydrofolate reductase), and MTHFD (methylenetetrahydrofolate dehydrogenase) to challenge the one-carbon metabolism; and the malic enzyme (Men). Knockdown of Men or enzymes related to one-carbon metabolism did not significantly affect tumor growth (Figure 2E). Regarding the PPP, knockdown of PGLS, but not G6PDH or 6PGDH, significantly affected tumor growth (Figure 2E). Therefore, the sole effect of PGLS does not allow us to discriminate whether only the PPP or the three metabolic pathways together contribute to NADPH production sustaining FA synthesis.

#### Metabolic regulators

Finally, we challenged the possible requirement of five regulators (HNF4, SREBP, Mondo, Mlx, and Branchless) previously shown to be directly or indirectly linked to energy metabolism. HNF4 adult fly mutants are glucose intolerant and defective in insulin secretion and mitochondrial functions.^48^ In *Drosophila*, SREBP has been shown to control phospholipid production in response to phosphatidylethanolamine levels.^49^ Mondo and Mlx form a bipartite transcription factor that modulates several metabolic pathways in response to dietary sugar.^50^^,^^51^ The fibroblast growth factor (FGF) ligand *Drosophila* homologue Branchless has been shown to control patterning and remodeling of the tracheal system.^52^ Knockdown of HNF4 or SREBP, but not Mondo or Mlx, strongly reduced tumor growth (Figure 2F). Finally, the moderate increase in tumor growth induced by Branchless knockdown (Figure 2F) suggests that the formation of additional trachea is not required.

These findings reveal that the growth of these tumors relies on several metabolic routes, including lactic fermentation and respiration. Intriguingly, these two metabolic pathways are not supposed to be concurrently active in the same cells, since the Crabtree effect reported glucose-induced repression of respiratory flux in tumor cells, while enhancing fermentation.^53^ Furthermore, both gluconeogenesis and FA synthesis are required for tumor growth, whereas the former is activated by glucose deprivation and the latter by glucose excess.^50^^,^^51^^,^^54^

### Single-cell RNA sequencing from tumors reveals distinct metabolic clusters

**Figure 3:**
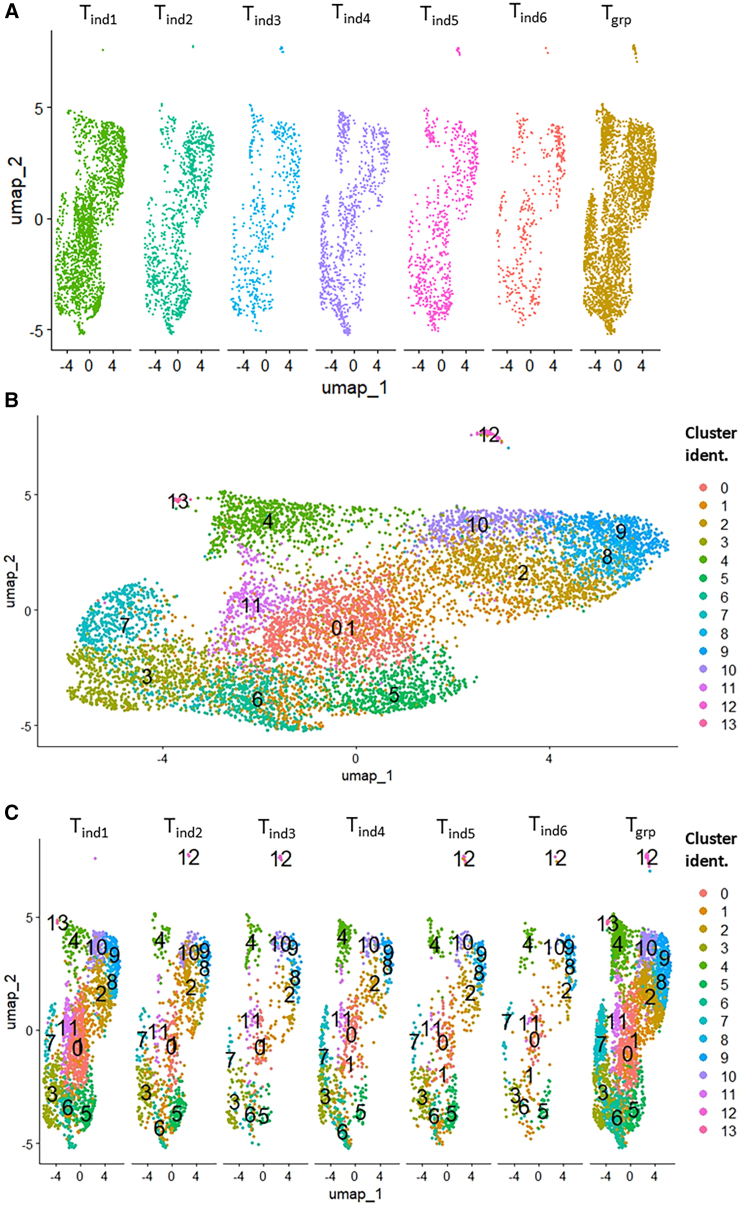
RNA-seq clustering analysis of midgut tumors (A) UMAP for each integrated individual sample reveal same levels of dispersion. (B) Clustering analysis resulting from all samples pooled together. (C) Iteration of clustering analysis for each individual sample displays that all clusters shown in (B) are present in each sample.

The unexpected great diversity of metabolic pathways required for the growth of these tumors, prompted us to perform single-cell RNA sequencing (RNA-seq) on midgut tumors. We successfully analyzed six well-delineated tumors individually and a group of six tumors together, each containing enough cells for clustering analyses (Figure S1). Using the Seurat R pipeline, we conducted quality control of each sample to verify mRNA diversity (Figure S2), and mitochondrial gene expression (Figure S3) as a marker of apoptosis to exclude dying cells for analysis. Comparison of UMAPs revealed strong similarities shared among all samples (Figure 3A). Since we recovered a moderate number of cells for individual tumors (Figure S1) and they all shared similar UMAP projections (Figure 3A), we pooled them to improve clustering resolution (Figure 3B). As a result, we identified 14 clusters, and back projection of these clusters for each sample showed that almost all of them can be detected in isolated tumors (Figure 3C). Individual counts for each cluster revealed that at least 12 of them were present in every tumor (Figure S4). Additionally, to prevent potential contamination, we checked common midgut cell markers^55^ and rat sarcoma (RAS) expression as a tumor-specific marker. Consistently, nearly all cells were positive for RAS (Figure S5), confirming their tumor identity. Regarding the visceral muscle signature, only one (*vein*) out of three markers could be detected in a few cells (Figure S5). For the enteroendocrine signature, two (*prospero* and *piezo*) out of 12 markers could be detected in some cells (Figure S5). For the enterocyte signature, while *myosin31DF* was detected in several cells, *nubbin* was present in a very few cells (Figure S5). Regarding the enteroblast signature, *escargot* was present in several cells, while *suppressor of Hairless* was present in very few cells (Figure S5). Importantly, for all these markers, positive cells were rarely seen in one same cluster, indicating marginal contamination by non-tumorous midgut cells.

**Figure 4:**
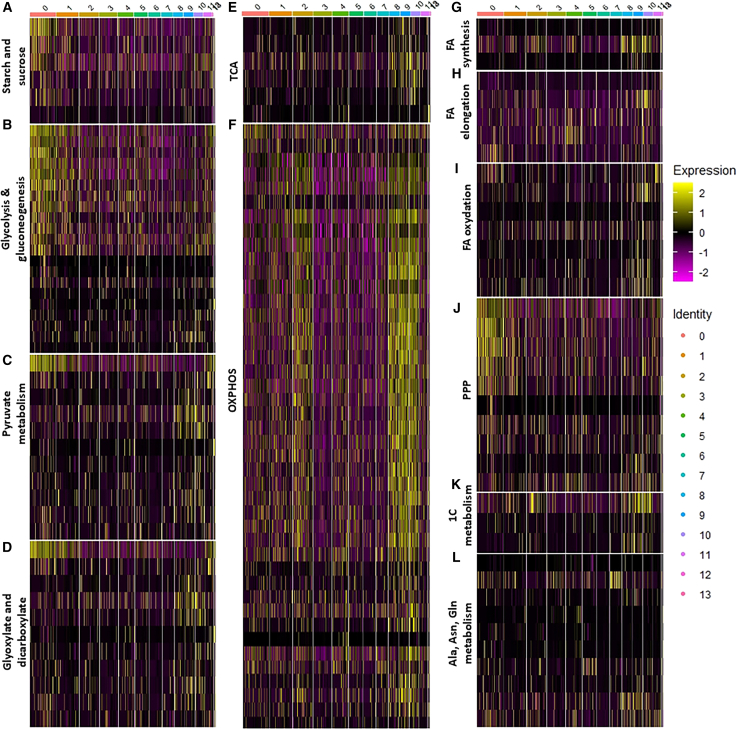
Transcript-based metabolic heterogeneity in tumors (A–L) Metabolic heatmaps of pooled tumor samples based on KEGG pathways related to sucrose and sucrose (A), glycolysis and gluconeogenesis (B), pyruvate metabolism (C) glyoxylate and dicarboxylate (D), TCA (E), OXPHOS (F), FA synthesis (G), FA elongation (H), FA oxidation (I), PPP (J), one-carbon metabolism (K), and amino acid metabolism (L).

Next, we performed gene ontology (GO) analysis based on biological processes (BPs), cellular components (CCs), and molecular functions (MFs). Despite insufficient cell count to retrieve relevant information for all clusters, GO analysis revealed specific functions for BP, CC, and MF categories in several clusters (Figures S6–S9). Focusing on the BP analysis, glycolytic- and respiratory-related processes were the main characteristics accounting for clusters 0 and 2, respectively (Figure S6). Consistently, this metabolic dichotomy was confirmed for CC and MF analyses (Figures S7 and S8). Based on the GO results, we selected metabolic-related genes from the respective KEGG pathway.^56^ This approach allowed us to attribute distinct metabolic functions to the most cell-abundant clusters (Figure S9). Based on the KEGG list, a general heatmap of metabolic genes was drawn (Figure S10). For clarity, these genes were grouped according to KEGG metabolic pathways (Figure 4). Cells from cluster 0 and, to a lesser extent, cluster 1 expressed high levels of glycolytic-related transcripts and low levels of respiratory-related transcripts, whereas clusters 2, 8, 9, and 10 showed opposite pattern (Figure 4). Clusters 3–7 expressed low levels of both glycolytic- and respiratory-related transcripts but exhibited other specific metabolic functions, including glutathione- and amino acid-related processes for cluster 3 and lipid-related processes for cluster 4 (Figures 4 and S9). Importantly, clusters 2, 4, and, in a lesser extent, 5 (respiring clusters) expressed cell-motility- and migration-related transcripts (Figure S9). Finally, focusing on a few genes retained for the metabolic screen, we confirmed cluster-independency between LDH and ETC components, and between FA synthesis and PEPCK2-dependent gluconeogenesis (Figure S11). Interestingly, GDH, ETCs components, ACC, and FASN1 were found in the same clusters. In summary, single-cell RNA-seq analysis revealed that individual tumors contain a diverse array of tumor cells characterized by specific metabolic signatures. Alternatively, the 14 identified clusters might be sub-clusters of the three major metabolic groups characterized by glycolysis, respiration, and FA synthesis.

### Metabolic diversity in late and early tumor phases

**Figure 5:**
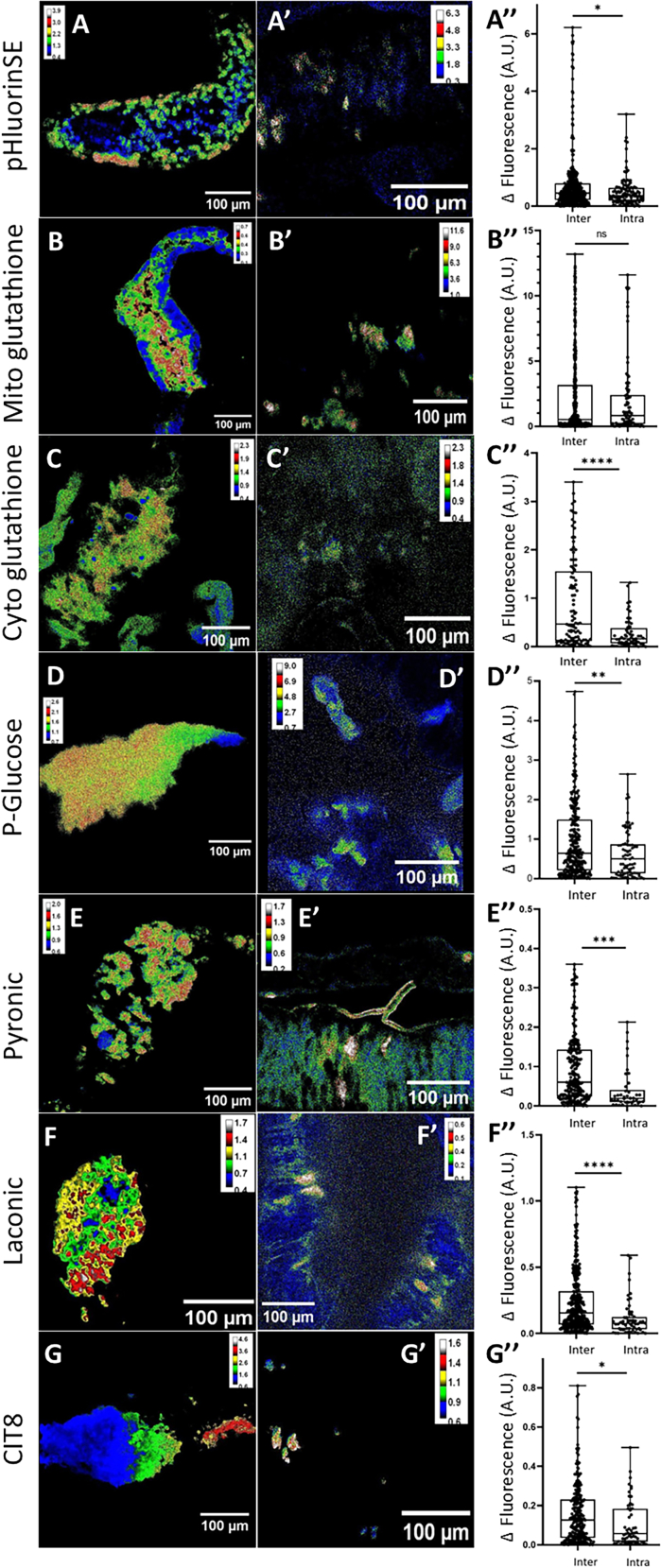
Metabolic sensor activities in tumors and clones (A–G) Genetically encoded sensors expressed in tumor cells only, to evaluate pH (A), mitochondrial oxidative state (B), cytosolic oxidative state (C), P-glucose levels (D), pyruvate levels (E), lactate levels (F), and citrate levels (G). Imaging were performed in late tumors (A–G) and early clones (A′–G′), the latter being used to compare fluorescence difference (Δ fluorescence) between cells of the same clone (intra) to cells from distant clones (inter) (A″–G″). For each graph, three independent series of at least 10 images were monitored the same day; each box represents the distribution (25%, 50%, and 75% quartiles). ∗*p* < 0.05,∗∗*p* < 0.0095, ∗∗∗*p* < 0.0002, and ∗∗∗∗*p* < 0.0001; Mann-Whitney test.

The critical need for ETCs (Figure 2B), the identification of respiratory-specific clusters (Figure 4), together with the fact that Branchless is dispensable (Figure 2F) suggest that mitochondrial activity varies within a given tumor. Consistently, MitoTracker staining revealed strong heterogeneity among tumor cells (Figure S12). Tumor cell heterogeneity was also observed for lipid (Figure S13) and ACC (antibody) staining (Figure S14), further supporting the metabolic diversity characterized via clustering analysis (Figure 4). Therefore, we wondered whether cellular diversity progressively develops during tumor progression or whether it is established early in somatically recombined clones. To investigate this issue, we recombined UAS-driven metabolic sensors for pH and redox states, and for metabolite levels with *UAS-RasV12*, and we removed the *UAS-GFP* transgene to prevent fluorescent interference. First, we studied single fluorescent sensors that monitor pH and redox states. At late stages (≥20 dpi), in well-delineated tumors, the pH sensor fluorescence revealed heterogeneity with a gradient ranging from an acidic core to a basic periphery (Figure 5A). At early stages (≤5 dpi), we analyzed intensity differences between cells from the same clone and cells from distinct clones (Figure 5A′). Importantly, the difference in fluorescence intensity was significantly higher between cells from distinct clones than within the same clone (Figure 5A″), indicating that differences in pH values exist early after clonal induction. To monitor the redox state, we used a glutathione sensor targeted to either the cytoplasm or the mitochondria. Similar to the pH pattern, the mitochondrial glutathione sensor revealed a core-to-periphery gradient in late tumors (Figure 5B). However, no significant fluorescence differences were observed when comparing cells from the same clone and cells from distinct clones at early stages (Figures 5B′ and 5B″). In contrast, when targeted to the cytoplasm, the glutathione sensor exhibited fluorescence differences both in late tumors and at early stages between cells from the same clone and cells from distinct clones (Figures 5C–5C″). To monitor metabolite levels, we made use of FRET-based sensors for P-glucose, pyruvate, lactate, and citrate. At late stages, in well-delineated tumors, all sensors exhibited marked fluorescence differences among tumor cells, although no typical pattern could be defined (Figures 5D–5G). Importantly, at early stages, significant differences in fluorescence were observed for each sensor between cells from the same clone and cells from distinct clones (Figures 5D′–5G′, 5D″–5G″). As a control, GFP-labeled late tumors and early clones exhibited regular and reproducible fluorescent variations without significant differences when comparing cells from the same clone and cells from distinct clones (Figure S15). Taken together, these findings indicate that late tumors show internal metabolic diversity, and that metabolic heterogeneity could be observed even at early stages between individual clones.

### Polyclonal tumors

**Figure 6:**
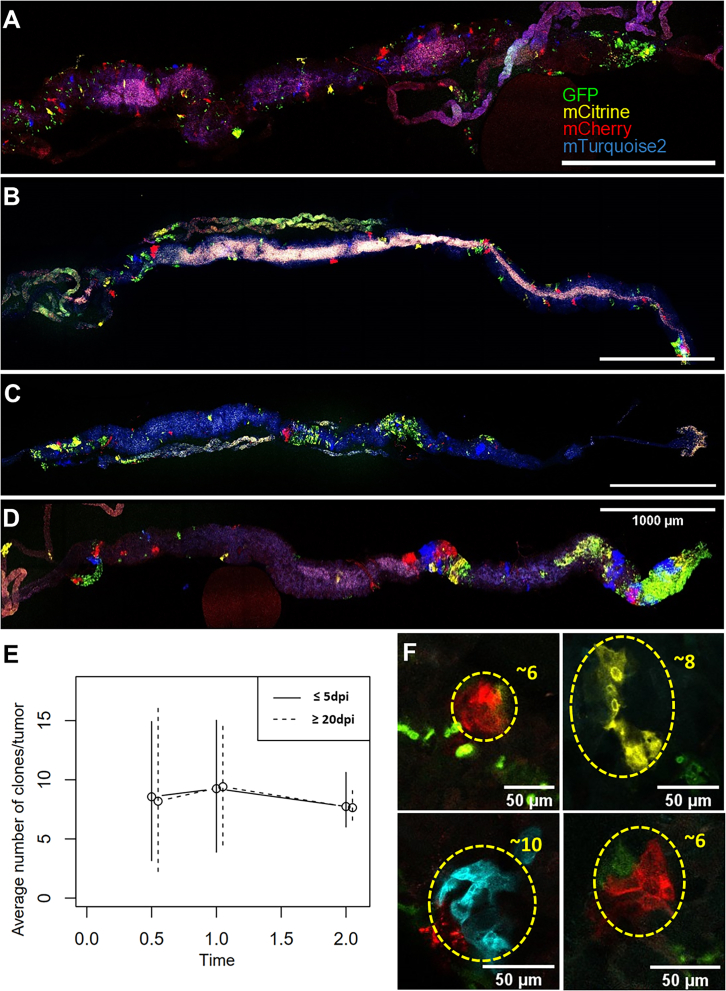
Florescent lineage analysis of tumors (A–D) Midguts (anterior to the right) at 3- (A), 10- (B), 14- (C), and 20 dpi (D) from 2 h tumor and fluorescence shuffling induction. (E) The figure represents the maximum likelihood estimate and the associated 95% confidence interval; images (≤5 dpi and ≥20 dpi) were analyzed for various induction times (*x* axis) to predict via a probabilistic model the average number of founder clones per tumor. (F) Clones at 4 dpi containing 6–10 cells as indicated. Fluorophores are shown in green (GFP), red (mCherry), blue (mTurquoise2), and yellow (mCitrine). Non-specific auto-fluorescence due to bacterial load in the midgut lumen is visible in pink (A and D), silver (B), and blue (C).

Considering on one hand the metabolic gene expression diversity in the tumor clusters and on the other hand, the metabolic heterogeneity in early and late tumor stages, we aimed to determine whether these tumors arise from a composite association of various founder clones. To investigate this issue, we employed the flybow strategy,^57^ which randomly marks clones with four different fluorophores. In this setting, both tumor induction and differential clone-labeling are induced by the same heat-shock performed on 2–3 day-old flies. Flybow-expressing digestive tracts were observed at various time points. As previously described,^37^^,^^38^ we frequently observed numerous individual clones at early stages progressing to more regionalized foci at intermediary stages and to well-delineated tumors at late stages (Figures 6A–6D). To evaluate the efficiency of fluorophore shuffling, we tested various induction times. The frequency of fluorophore appearance was calculated by counting the occurrences of each color in each image dataset. For a 30-min induction, GFP was the most abundant fluorophore, while the other three fluorophores were dramatically underrepresented (Figure S16), consistent with the fact that non-induced flybow rearrangement results in GFP labeled clones.^57^ Increasing the induction time to one and two hours markedly equalized the relative occurrence of each fluorophore to a more even distribution, although GFP remained overrepresented (Figure S16). Detailed analysis evaluated the relative fluorophore proportions in both clones and tumors at ≤5 dpi and ≥20 dpi for each induction time (Figures S17 and S18). The distribution revealed that clones are more abundant at ≤5 dpi than at ≥20 dpi (Figure S17), suggesting that clones are eliminated as the days post-induction increase. Conversely, the number of tumors was very low at ≤5 dpi and strongly increased at ≥20 dpi (Figure S18). Notably, tumors expressing a single fluorophore were always GFP positive (Figure S18), reflecting the predominance of GFP among observed fluorophores (Figure S16). The presence of tumors at early stages and the consistent GFP positivity in mono-labeled tumors strongly support their multi-clonal origin. Based on the relative distributions of fluorophores in both clones and tumors, we constructed a probabilistic model to estimate the theoretical number of founder clones required to form a tumor. The best-fitting model predicted that tumors typically result from the association of approximately eight initial clones (Figure 6E). Importantly, we could observe clones containing up to 10 cells at 4 dpi indicating that at least three cell division events may happen during this period (Figure 6F). At such rate, a single clone may have undergone 15 division events at 20 dpi, resulting in a tumor theoretically containing 32,768 cells, thereby excluding that, as a consequence of slow division rate, tumors would be observed solely when several clones assemble together. Taken together, these findings provide evidence that these intestinal tumors invariably arise from an association between several distinct founder clones.

### Cell motility

**Figure 7:**
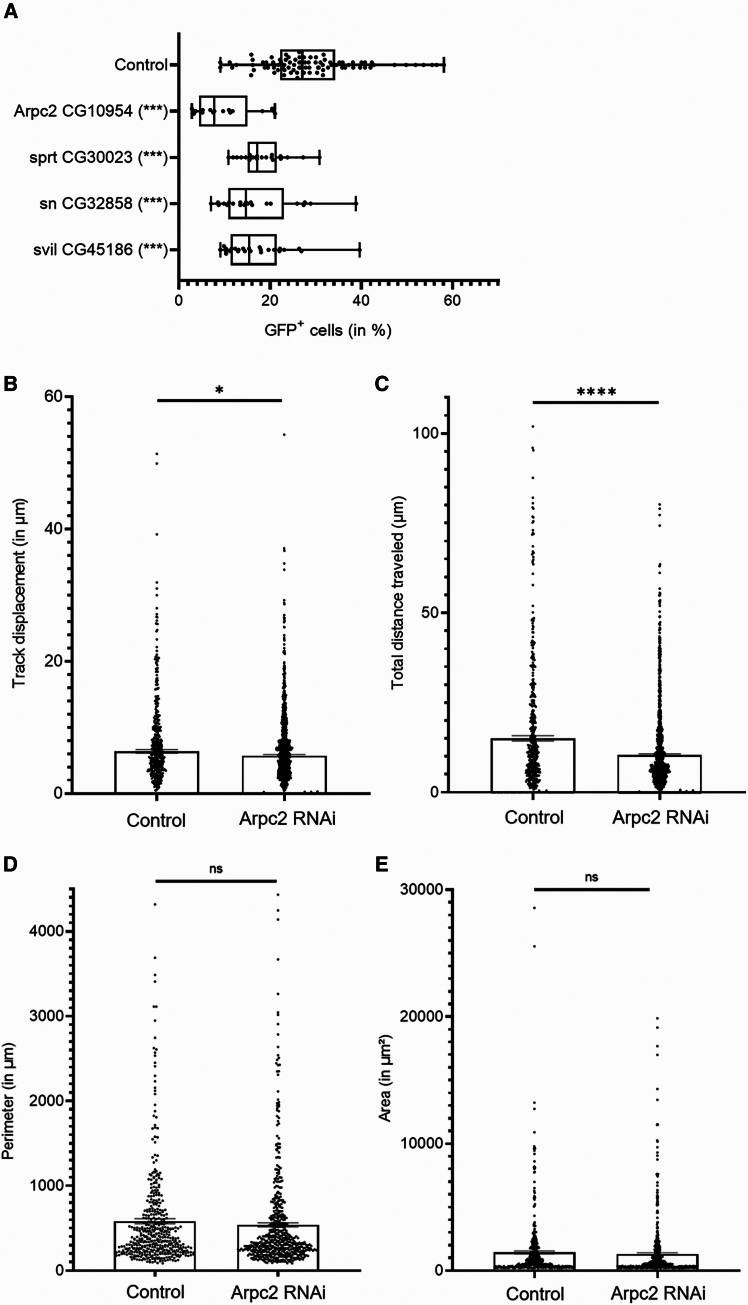
Motility of tumor cells (A) FACS measurement of GFP^+^, tumor cells in 21-dpi midguts expressing RNAi to various motility-related. (B and C) Overnight track analysis of GFP^+^ cells displacement (B) and total distance traveled (C) from tumor cells either control or expressing an RNAi to Arpc2. (D and E) Perimeters (D) and areas (E) of GFP^+^ tumor cells estimated from overnight movies in control and Arpc2-RNAi expressing cells. For FACS measurements (A), each dot is a replicate containing five midguts; box represents the distribution (25%, 50%, and 75% quartiles); *p* values were calculated from a generalized linear model (overdispersed binomial), the control being considered as the intercept of the model. *p* values were corrected for multiple testing by the Holm-Bonferoni method; ∗∗∗ < *p* = 0.001 < ∗∗ < *p* = 0.01 < ∗ < *p* = 0.05 < (ns). For motility (B, C, D, and E), each dot represents displacement of one cell followed for at least 2 h 30 min (i.e., 7 consecutive time points). ∗*p* < 0.05 and ∗∗∗∗*p* < 0.0001; unpaired t test. Data are represented as mean ± SD.

The association of clones to form a delineated tumor, together with the metabolic diversity in distant clones, suggests that cell mobility must be required for tumor progression. To investigate this issue, we generated fly lines to knock down *Arpc2*, *sprt*, *sn*, and *svil*, four genes required for cell motility.^58^ A significant reduction in tumor growth was observed for each gene knockdown, with *Arpc2* resulting in the strongest suppression (Figure 7A). Focusing on *Arpc2*, we monitored overnight tumor cell movements from *ex vivo* midgut tracts (Videos S1 and S2). Film visualization suggested three potential modes influencing cell displacements: (1) peristalsis, (2) spontaneous delamination within the gut lumen, and (3) *bona fide* cell displacement within the epithelium. Comparing flybow tumorous cell movement using the TrackMate extension on Fiji revealed a significant reduction in cell track displacement in *Arpc2* knockdown tumors (Figure 7B), and an even more significant reduction in total distance traveled (Figure 7C). Interestingly, no significant differences were observed in cell perimeters and areas between the two conditions (Figures 7D and 7E), ruling out a potential subsequent effect of cell size impacting cell mobility. Altogether, these results indicate that cell motility is required for tumor formation.

Video S1. Cell displacements in control tumors Overnight live imaging of adult *Drosophila* digestive tract three days past eclosion, which contains tumors expressing the flybow cassette (1 image/22 min; films at 7 fps).

Video S2. Cell displacements in Arpc2 knockdown tumors Overnight live imaging of adult *Drosophila* digestive tract three days past eclosion, which contains tumors expressing the flybow cassette and Arpc2 RNAi (1 image/22 min; films at 7 fps).

## Discussion

In this study, we show that our *Drosophila* tumor model exhibits metabolic changes typically found in mammalian cancers, including the requirements of (1) both lactate metabolism and functional ETCs^32^^,^^33^; (2) an unconventional Pyk^59^; (3) GlyS that catalyzes glycogen synthesis^60^; (4) mitochondrial glutamine consumption, but not OAA synthesis catalyzed by Pcb, to sustain TCA anaplerosis^32^^,^^42^; and (5) enzymes of gluconeogenesis.^61^ Based on these metabolic needs, further experiments provide evidence that despite their identical genetic background, these intestinal tumors comprise cell populations with different metabolic specificities and are always polyclonal.

According to the Crabtree effect,^53^^,^^62^ the dual requirement of lactate metabolism and functional ETCs suggests the presence of different cell populations performing either lactate fermentation or mitochondrial respiration. Since the *Drosophila* genome encodes a unique LDH working in both directions, we cannot determine whether it results in the production of lactate or pyruvate, the latter being possibly consumed via respiratory flux. A study reported strong lactate dependency in lung tumors^63^^,^^64^ and another one that ablation of ETC components in a lung tumor mouse model suppresses tumor growth.^65^ In mammalian tumors, this dual need has been reconsidered and investigated regarding tumor architecture.^32^^,^^33^^,^^66^ Cells close to vessels receive high amounts of oxygen, while those located far away are in severe hypoxic conditions. The latter may produce and secrete lactate, which is taken up by the mitochondria of the former or by host-associated fibroblasts within the tumor microenvironment.^67^ In our tumor model, the strong effects of LDH and ETC knockdowns raise the possibility that LDH is required in both fermenting and respiring cell types since the moderate effects of PFK and Pyk knockdowns suggest that glycolysis is unlikely to sustain both lactate biogenesis and mitochondrial respiration. However, we cannot exclude alternative pathways, as reported for glycolysis in tumor cells *ex vivo*^68^ or direct fructose oxidation bypassing the PFK1-dependent preparatory phase of glycolysis.^69^ Additionally, it has been shown that in hypoxic condition, a glutamine reductive metabolism, via a reverse canonical TCA route, provides citrate for FA biogenesis, independently of ETCs activity.^70^^,^^71^ Importantly, the RNA-seq identification of fermenting and respiring clusters, as well as the unequal mitochondrial staining suggest differential respiration efficiency among tumor cells. Additionally, the knockdown of Bnl—an FGF orthologue required for intestinal tracheal rearrangement^52^—showing no effect on tumor growth indicates that the formation of additional trachea is dispensable. Altogether, these findings strongly support that these tumors comprise distinct cell populations fulfilling either fermenting or respiring functions.

The requirement of transaminases, glutamine metabolism, FA synthesis, and gluconeogenesis support the need for a TCA anaplerotic/cataplerotic axis. In cancer cells, anaplerosis to replenish with TCA intermediates, typically depends on glutamine consumption.^42^^,^^65^ Gluconeogenesis, a cataplerotic pathway resulting in OAA deprivation, recruits PEPCK, FBP, and G6P to bypass irreversible glycolytic reactions. In tumor cells, this pathway does not obligatory result in glucose production as in starved hepatic cells, but may feed glycerol synthesis or PPP.^61^ However, since the knockdown of GPO1—catalyzing glycerol-3P synthesis—had no effect, while the knockdown of only one of the three PPP-related enzymes resulted in a significant tumor growth reduction, we cannot precisely determine the eventual fates of this cataplerotic pathway. Cataplerosis may also work to sustain FA synthesis, via citrate efflux from mitochondria. Interestingly, GDH, ETCs components, and FA synthesis enzyme segregate in the same clusters, suggesting that GDH-dependent anaplerosis compensates for citrate mitochondrial efflux. Importantly, while both FA synthesis and gluconeogenesis are cataplerotic processes, they are unlikely to operate concurrently in the same cells, since the former is activated by glucose excess, while the latter is activated by glucose deprivation.^50^^,^^51^^,^^54^ Consistently, the only PEPCK2-expressing cluster excluded the GDH/ETC/FA synthesis axis. Additionally, lipid accumulation and ACC staining were not uniformly detected in the entire tumor, further supporting a metabolic patchwork architecture.

In the cytoplasm, citrate cleavage to OAA and acetyl-CoA is catabolized by ACLy, acetyl-CoA is carboxylated by ACC in malonyl-CoA, which is used by FASN to build up LCFAs. That ACLy and ACC knockdown strongly suppressed tumor growth whereas, the one of FASN tended, although not significantly, to reduce tumor growth may result from malonyl-CoA levels, which in addition to sustaining LCFA synthesis also inhibits their oxidation.^72^ ACLy or ACC knockdown depletes malonyl-CoA, whereas FASN knockdown likely increases malonyl-CoA amounts, resulting in opposite effects on LCFA β-oxidation. This requirement questions whether the production of LCFA is critical for tumor growth, as proposed for several human cancers.^73^ Here, we found that none of the enzymes potentially involved in TAG and/or phospholipid synthesis are required, possibly because of gene redundancy. Nonetheless, SREBP that regulates phospholipid synthesis,^49^ but not Mondo/Mlx that stimulates FA and TAG synthesis,^50^^,^^51^ is critically required for tumor growth. Therefore, phospholipid synthesis is likely critical for the growth of these tumors to support the great demand for cell membranes.

Based on the Crabtree effect,^31^ our findings indicate that the metabolic pathways operating in our tumor model are unlikely concurrently active in the same cells. The sensor patterns, which vary depending on the targeted metabolite, suggest a complex metabolic-based tumor architecture organized as a discontinuous patchwork of several metabolic subdomains, further supported by the RNA-seq identification of multiple metabolic-specialized clusters. The existence of metabolically active subdomains that cooperate has also been characterized in mammalian cancers.^1^^,^^67^^,^^74^^,^^75^ Importantly, we could never observe monoclonal tumors in our fluorescent cell lineage dataset. However, since we noticed that, in early stages, clones proliferate almost at one division per day, monoclonal tumors should be observed after three weeks. Rather, our experimental-based probabilistic model predicts that tumors derive from several clones ranging from six to twelve founders, an order of magnitude reminiscent of the RNA-seq clustering for each individual tumor. Furthermore, congruent to the association of aerobic-metabolism with metastatic dispersion in mammalian tumor cells,^76^ we also found a higher expression of motility-related genes in respiring clusters. However, even though these tumors are non-metastatic,^77^ genes involved in cell motility are required for tumor growth. Cell motility might be required for founder clones gathering to form a functional tumor. Potentially, founder clones provide complementary cellular functions as visualized by different metabolic specificity soon after tumor induction. The fact that metabolic genes constitute a key parameter to discriminate clusters argue for a link between metabolism and polyclonality, which remains to be formally demonstrated. Nonetheless, it is unlikely that the metabolic specificity of each founder clone persists during tumor progression since the metabolic and lineage architectures differ in the eventual tumor. Deciphering the underlying mechanisms will require further investigations to deeply characterize the cellular functions of founder clones and their dynamics during the tumor formation process.

Our study aligns with experimental models of intestinal tumors. In chimeric mice carrying two distinct tumor-promoting mutations, the statistical occurrence of chimeric tumors supports their non-monoclonal origin as long as initiating clones are in close proximity.^20^ Feeding carcinogens also induced the formation of intestinal tumors of polyclonal origin in mice.^21^ Interestingly, it has been reported that oncogenic transformation is facilitated near a progenitor tumor clone leading to the formation of polyclonal tumors.^27^ The polyclonal architecture of mouse intestinal tumors was also reported both in the early adenoma and late carcinoma stages.^22^ Studies on another mouse intestinal model revealed that colon tumors are monoclonal in origin but eventually assemble in polyclonal tumors, while small intestine tumors are of polyclonal origin.^23^ In *Drosophila*, a seminal study reported that clones in imaginal discs carrying a loss-of-*scribble* mutation together with ectopic expression of the RasV12 oncogene resulted in metastatic tumors.^78^ Strikingly, further investigations revealed that after the somatic recombination generating mutant clones, if one of the daughter cells carries the *scribble* mutation, while the other one expresses RasV12, the resulting couple of cells may progress to an invasive tumor.^79^ Taken together, these studies strengthen the notion that a cancer is made of different cell types that cooperate to favor tumor progression. Therefore, considering the previously described advantage of cell diversity, the probability of cancer initiation must take into account the statistical frequency of primary mutations, the cell division rate of the host tissue, the exposure to mutagen agents, and the advantage of initial diversity for cancer progression.^80^ While tumor growth is defined as the increase in cell mass, cancer progression corresponds to the step-by-step transformation of the cell status. Therefore, the onset of complementary functional interactions between cancer cells, but also with host cells from the microenvironment must be considered as a critical step of cancer progression, irrespective of a monoclonal or polyclonal origin. Understanding these processes that stem on tumor initiation and progression are crucial for establishing appropriate anticancer strategies to each type of cancers.

### Limitations of the study

Here we provide evidence (1) that these intestinal tumors are always polyclonal; (2) that the rate of cell division is not limiting; (3) that the major cell clustering of these tumors is essentially metabolic-based; (4) that metabolic heterogeneity already exists between founder clones; and (5) that cell motility genes are required for tumor growth. Nonetheless, the *Drosophila* genetic approach does not allow for following the metabolic fate of the founder clones to determine whether they are at the origin of the different metabolic clusters within the resulting tumors. These issues should be addressed in future studies investigating the dynamics of the metabolic fates of the founder clones in relation to their motility during the process of tumor progression. Furthermore, to sustain the formation of these obligate polyclonal tumors, numerous clones must be generated simultaneously, raising the question of whether such a tumor clone frequency may spontaneously happen. However, old *Drosophila* flies have been reported to develop—at very low frequency—spontaneous intestinal tumors, for which polyclonality should be investigated.^81^ Alternatively, immortalized clones may persist, being silent over a long period, while additional transformed clones may arise in their vicinity to elicit tumor formation, in particular in long-lived metazoans. In summary, to assess the risk of polyclonal CRC in human, it is critical to evaluate the putative frequency of transformed clones present simultaneously in a given region of the digestive tract, taking into account cell turnover rate, exposure to mutagens, and individual patient genomes.

## Resource availability

### Lead contact

Requests for further information and resources should be directed to and will be fulfilled by the lead contact, Jacques Montagne (jacques.montagne@ens-lyon.fr).

### Material availability

No reagents and no original *Drosophila* lines have been generated for this study. All the fly strains (Figures 2, 5, and 7) were generated by recombining and/or combining lines from stock centers or from generous gift of A. Casali, I. Salecker, or P.Y. Placais. The recombined *Drosophila* stocks will be kept in our collections for a few months, but their availability also requires the agreement of the owners of the initial lines.

### Data and code availability

• Sequencing data have been deposited at GEO NCBI (https://www.ncbi.nlm.nih.gov/geo/) and are publicly available as of the date of publication with the accession number Series GSE271736.
• All original code has been deposited at Github (https://www.github.com) and is publicly available at https://github.com/Pdelam/Delamotte-et-al.-2024.git as of the date of publication.
• Unprocessed data (microscopy, cytometry files, films, etc.) reported in this paper will be shared by the lead contact upon request.

## Acknowledgments

We wish to thank A. Casali, I. Salecker, and P.Y. Placais for stocks and stimulating discussions; nonprofit funding to J.M. (Fondation ARC contre le Cancer PJA 20181208078 and ARCPJA2022060005236, 10.13039/501100004099French League Against Cancer M27218) and P.D. (fellowships MENRT 2020-110 and French League Against Cancer IP/SC-178135); Céline Hernandez and Delphine Naquin for helpful discussions on RNA-seq analyses; the sequencing and bioinformatics expertise of the I2BC High-throughput sequencing facility, supported by France Génomique (funded by the French National Program “Investissement d’Avenir” ANR-10-INBS-09); and the Imagerie-Gif Core facility for technical assistance (funded by ANR: ANR-11-EQPX-0029/Morphoscope, ANR-10-INBS-04/FranceBioImaging, ANR-11-IDEX-0003-02/Saclay Plant Sciences).

## Author contributions

P.D., M.P., Y.J., and J.M. performed experiments; P.D., A.L.R., and J.M. analyzed the data; P.D., A.L.R., and J.M. wrote the manuscript; J.M. conceived the study and provided financial support.

## Declaration of interests

The authors declare no competing interest.

## STAR★Methods

### Key resources table

**Table:** 

REAGENT or RESOURCE	SOURCE	IDENTIFIER
**Antibodies**		

Polyclonal rabbit Anti ACC	Parvy et al.^43^	N/A
Goat anti-rabbit Alexa 647	Thermo Fisher Scientific	Cat. No. A-21245 RRID: AB_2535813

**Chemicals, peptides, and recombinant proteins**		

Trypsin	PAN BIOTECH	P10-022100
Collagenase type XI	Sigma-Aldrich	Ref. C7657
Hoechst 33342	Thermo Fisher Scientific	Cat. No. H3570
MitoTracker	Thermo Fisher Scientific	ref. M22426
LipidTox	Thermo Fisher Scientific	ref. H34477

**Deposited data**		

Single-cell RNA sequencing results	GEO NCBI (https://www.ncbi.nlm.nih.gov/geo/)	Accession number: Serie GSE271736
ImageJ macro, R scripts and raw data	Github (https://www.github.com)	Accession: https://github.com/Pdelam/Delamotte-et-al.-2024.git

**Experimental models: Organisms/strains**		

*Drosophila melanogaster*, UAS-pH sensor	Mahon et al.^82^	N/A
*Drosophila melanogaster*, UAS-glutathionne sensors	Albrecht et al.^83^	N/A
*Drosophila melanogaster*, UAS-glucose sensor	Miyamoto et al.^84^	N/A
*Drosophila melanogaster*, UAS-pyruvate sensor	Arce-Molina et al.^85^	N/A
*Drosophila melanogaster*, UAS-metabolic sensors	Hudry et al.^86^	N/A
*Drosophila melanogaster*, Flybow	Hadjieconomou et al.^57^	N/A
*Drosophila melanogaster*, Line 1	Martorell et al.^37^	Fly genetics
*Drosophila melanogaster*, Line 2	Martorell et al.^37^	Fly genetics
*D. melanogaster*, RNAi to CG7176 (IDH)	BDSC	41708
*D. melanogaster*, RNAi to CG17725 (pepck1)	BDSC	65087
*D. melanogaster*, RNAi to CG1444 (VLC 3-oxoacyl-CoA reductase)	VDRC^87^	40949
*D. melanogaster*, RNAi to CG1516 (Pyruvate carboxylase)	VDRC	105936
*D. melanogaster*, RNAi to CG1640 (Alanine transaminase)	VDRC	32681
*D. melanogaster*, RNAi to CG1707 (Glyoxalase I)	VDRC	26832
*D. melanogaster*, RNAi to CG1946 (Diacylglycerol acyltransferase)	VDRC	108495
*D. melanogaster*, RNAi to CG2964 (Pyruvate kinase/PK)	VDRC	422933
*D. melanogaster*, RNAi to CG3209 (GPAT4)	VDRC	100728
*D. melanogaster*, RNAi to CG3350 (mlx/bigmax)	VDRC	110630
*D. melanogaster*, RNAi to CG3415 (Mfe2)	VDRC	108880
*D. melanogaster*, RNAi to CG3523 (FASN1)	VDRC	108339
*D. melanogaster*, RNAi to CG3612 (ATPsynthase alpha chain)	VDRC	34664
*D. melanogaster*, RNAi to CG3724 (phosphogluconate DH)	VDRC	100269
*D. melanogaster*, RNAi to CG3861 (knd/Citrate synthase)	VDRC	107642
*D. melanogaster*, RNAi to CG4001 (6-phosphofructokinase)	VDRC	105666
*D. melanogaster*, RNAi to CG4067 (pug/folate metabolism)	VDRC	26384
*D. melanogaster*, RNAi to CG4233 (Got 2)	VDRC	106120
*D. melanogaster*, RNAi to CG4608 (branchless)	VDRC	101377
*D. melanogaster*, RNAi to CG4753 (Agpat4)	VDRC	1730
*D. melanogaster*, RNAi to CG5320 (Glutamine dehydrogenase)	VDRC	109499
*D. melanogaster*, RNAi to CG5362 (Mdh1/Malate DH 1)	VDRC	110604
*D. melanogaster*, RNAi to CG5508 (GPAT1)	VDRC	1316
*D. melanogaster*, RNAi to CG6904 (Glycogen synthase/GlyS)	VDRC	35136
*D. melanogaster*, RNAi to CG7010 (Pyruvate DH)	VDRC	107209
*D. melanogaster*, RNAi to CG7069 (Pyruvate kinase)	VDRC	101116
*D. melanogaster*, RNAi to CG7070 (pyruvate kinase)	VDRC	49533
*D. melanogaster*, RNAi to CG7176 (Isocitrate DH)	VDRC	100554
*D. melanogaster*, RNAi to CG7361 (RFeSP)	VDRC	330703
*D. melanogaster*, RNAi to CG7362 (Pyruvate kinase/PK)	VDRC	104218
*D. melanogaster*, RNAi to CG7433 (γ-aminobutyric acid transaminase)	VDRC	110468
*D. melanogaster*, RNAi to CG7560 (Methylenetetrahydrofolate reductase)	VDRC	103792
*D. melanogaster*, RNAi to CG7998 (Mdh2)	VDRC	101551
*D. melanogaster*, RNAi to CG8256 (Glycerol-3-phosphate DH)	VDRC	110608
*D. melanogaster*, RNAi to CG8322 (ACLY/ATPCL)	VDRC	30282
*D. melanogaster*, RNAi to CG8430 (Got 1)	VDRC	108247
*D. melanogaster*, RNAi to CG8522 (SREBP)	VDRC	37641
*D. melanogaster*, RNAi to CG8657 (diacylglycerol kinase ε)	VDRC	4659
*D. melanogaster*, RNAi to CG8709 (Lipin PAP)	VDRC	107707
*D. melanogaster*, RNAi to CG8808 (Pdk1)	VDRC	37968
*D. melanogaster*, RNAi to CG9244 (mitochondrial aconitase)	VDRC	12455
*D. melanogaster*, RNAi to CG9310 (dHNF4)	VDRC	12692
*D. melanogaster*, RNAi to CG10120 (Malic enzyme)	VDRC	104016
*D. melanogaster*, RNAi to CG10160 (LDH/Imp-L3)	VDRC	110190
*D. melanogaster*, RNAi to CG10924 (pepck2)	VDRC	13929
*D. melanogaster*, RNAi to CG10954 (Arpc2)	VDRC	104396
*D. melanogaster*, RNAi to CG11015 (COX5B)	VDRC	105769
*D. melanogaster*, RNAi to CG 11198(ACC)	VDRC	108631
*D. melanogaster*, RNAi to CG11661 (E1 a-ketoglutarate complex)	VDRC	107713
*D. melanogaster*, RNAi to CG12529 (glucose-6-phosphate DH)	VDRC	101507
*D. melanogaster*, RNAi to CG14740 (Citrate synthase)	VDRC	100676
*D. melanogaster*, RNAi to CG15400 (G6P/Glu6P Phosphatase)	VDRC	330197
*D. melanogaster*, RNAi to CG17246 (Succinate DH)	VDRC	330053
*D. melanogaster*, RNAi to CG17333 (6-phosphogluconolactonase)	VDRC	100734
*D. melanogaster*, RNAi to CG17725 (pepck1)	VDRC	20529
*D. melanogaster*, RNAi to CG17725 (pepck1)	VDRC	50253
*D. melanogaster*, RNAi to CG18362 (ChREBP/mondo)	VDRC	109821
*D. melanogaster*, RNAi to CG18466 (Nm*D. melanogaster*c)	VDRC	110198
*D. melanogaster*, RNAi to CG30023 (Sprite)	VDRC	107873
*D. melanogaster*, RNAi to CG31692 (fbp/Fruct 1–6 Phosphatase)	VDRC	108554
*D. melanogaster*, RNAi to CG32858 (Singed)	VDRC	105747
*D. melanogaster*, RNAi to CG45186 (Supervilin)	VDRC	43583

**Software and algorithms**		

Cytexpert	Beckman Coulter	https://www.beckman.fr/flow-cytometry/
Leica Application Suite X (3.5.6)	Leica Microsystems	http://www.leica-microsystems.com/
Graph Pad Prism	Graph Pad	https://www.graphpad.com/
Microsoft Excel	Microsoft	https://products.office.com/en-gb/excel
ImageJ	National Institute of Health	https://products.office.com/en-gb/excel
R v 4.1	The R Project	https://www.r-project.org/

**Other**		

Leica inverted DMi 6000	Leica Microsystems	http://www.leica-microsystems.com/
CytoFLEX S	Beckman Coulter	https://www.beckman.fr/flow-cytometry/
NextSeq 2000 Instrument	Illumina	https://www.illumina.com/

### Experimental model

#### Fly genetics and husbandry

To generate tumors, *yw,HS-flp*; *esg-gal4*,*UAS-GFP*; *FRT82B*,*Tub-Gal80* (Line 1) and *yw,HS-flp*; *UAS-Ras*^*V12*^; *FRT82B*,*Apc2*^*N175K*^*,Apc*^*Q8*^ (Line 2)^37^ were crossed together. For knockdowns and metabolic investigations in tumors RNAi lines and UAS-driven metabolic sensors were recombined with the second chromosome carrying *UAS-Ras*^*V12*^. For cell lineage the *UAS-GFP* was replaced by the flybow cassette and the HS-mflp5 transgene was recombined with the *yw,HS-flp* transgene. Flies were maintained on our standard media.^40^ Following reviewer’s request, we have included a genetic and physiological section concerning the *Drosophila* model in the supplemental information (Methods S1).

### Method details

#### Flow cytometry

Experiments were performed on both male and female flies, which were collected and put together, with 30–40 flies per vial. Tumor induction was performed by a 1 h 15 min heat-shock on 2–3 days newly emerged adult flies. Flies kept on standard medium at 25°C were transferred in new vials three times a week; at 21 dpi, tumor growth was measured by cytometry as previously described.^38^^,^^39^ For each measurement, 5 midguts (with their Malpighian tubules) of either male or female flies were put together, and a genotype was considered complete after 10 measurements of males and females.

#### Single-cell RNAseq

Tumorous flies were heat-shocked at 37°C for 2-h 2–3 days after adult eclosion. At least 21-dpi flies were selected based on GFP fluorescence through the abdomens. Tumors were dissected on ice in 1X Ringer buffer, rinsed twice in ice-cold 1X Ringer baths, and the extracellular matrix was digested by gentle pipetting for 3–5 min with 20μL 5X Trypsin (PAN BIOTECH P10-022100) twice diluted. Efficient digestion was promptly checked under a fluorescent dissecting microscope. Consequently, for each sample, 1X Ringer buffer was added up to 1.5 mL and each tube was filtered through 30μm filters (Sysmex CellTrics). Tubes were centrifuged for 4 min at 1000G, at 4°C; 1 mL of supernatant was discarded and the same volume of fresh 1X Ringer buffer was added. A second centrifugation (4 min, 1000G, 4°C) was done, and after careful supernatant removal, pellets were gently resuspended in 30μL 1X Ringer buffer for sequencing. The whole sample preparation procedure did not exceed 20 min. Cell suspension was loaded into the Chromium Controller using a Chromium Next GEM Single Cell 3′ v3.1 kit (10x Genomics) to generate a droplet emulsion. cDNA was purified and libraries were prepared, according to the manufacturer’s recommendations. Single-cell libraries were sequenced on a NextSeq 2000 Instrument (Illumina) using a P2-100 cycles kit. FastQ files were analyzed using the Cell Ranger software (10X Genomics, version 6.0.1), including alignment, filtering and quantitation of reads on the *Drosophila* genome r6.42 and generation of feature-barcode matrices. **All the sequencing libraries obtained are available on GEO NCBI (**https://www.ncbi.nlm.nih.gov/geo/) **to** **GSE271736**.

We used the Seurat R pipeline^88^ for sample quality control and analysis. The R script used to evaluate sample quality, generate clusters, UMAPs, heatmaps, and GO analysis is available at: https://github.com/Pdelam/Delamotte-et-al.-2024.git.

#### Live imaging

Images were acquired with a Leica inverted DMi 6000 (**lasers** 405 nm diode (Leica, 50 mW), a 440 nm pulsed diode LDH-P-C-440 B (0.8 à 4.0 mW @40MHz) (Picoquant) and a white light laser 2 (80 MHz, from 470 to 670 nm) + pulse-picker. The software used for acquisition was Leica Application Suite X (3.5.6), and images were processed using ImageJ. Laser parameters and gating settings are detailed in Table below.

**Table:** 

	Channels								Analysis
Usage	Ch1 λexc	Ch1 λem	Ch2 λexc	Ch2 λem	Ch3 λexc	Ch3 λem	Ch4 λexc	Ch4 λem	
Flybow	585	595; 701	515	525; 550	440	451; 480	490	500; 512	Channel merge; regular fluorescence, brightness/contrast adjust if necessary
mcd8-GFP	488	500; 550	NA	NA	NA	NA	NA	NA	Regular fluorescence, brightness/contrast adjust if necessary
pHluorinSE	405	500; 550	485	500; 550	NA	NA	NA	NA	Threshold, channel1/channel2, LUT
mito glutathione	405	500; 550	488	500; 550	NA	NA	NA	NA	Threshold, channel2/channel1, LUT
cyto glutathione	405	500; 550	488	500; 550	NA	NA	NA	NA	Threshold, channel2/channel1, LUT
P-glu	440	460; 510	512	525; 650	440	525; 650	NA	NA	Threshold, channel2/channel1, LUT
pyronic	440	460; 510	512	525; 600	440	525; 600	NA	NA	Threshold, channel2/channel1, LUT
laconic	440	460; 510	512	525; 600	440	525; 600	NA	NA	Threshold, channel2/channel1, LUT
CIT8	440	460; 510	512	525; 650	440	525; 650	NA	NA	Threshold, channel2/channel1, LUT
MitoTracker	405	420; 500	488	498; 570	640	650; 790	NA	NA	Regular fluorescence, brightness/contrast adjust if necessary
LipidTOX	405	420; 500	488	498; 570	640	650; 790	NA	NA	Regular fluorescence, brightness/contrast adjust if necessary
ACC	405	415; 461	488	498; 570	568	610; 790	NA	NA	Regular fluorescence, brightness/contrast adjust if necessary

For live-imaging, 5–7 digestive tracts were aligned on an ibidi dish as detailed.^89^ Slight adjustments of this live-imging protocol were made: instead of setting midguts on agar pads, 7 midguts were centered on the ibidi dish, aligned next to each other, and embedded in 0.5% low-melting agar to facilitate imaging. Additionally, for overnight imaging, ibidi dishes were filled with complete culture medium reaching the upper rim of the dish and a wet Kimtech tissue was stuck on the upper lid of the dish to prevent liquid evaporation. For sensor monitoring, whole experimental fields were acquired at 20X by a 20 image-mosaic with a 5–8 z stack. For sensor analysis, raw images were i) converted to a macro-compatible format, ii) converted to a 32-bit format, and iii) stacked, and an automatic threshold was applied. Depending on the sensor, the resulting image was calculated by dividing the appropriate channels, and for visuals, a 5-ramp LUT and scales were applied. Raw and processed images are available on demand, and macro scripts are available at: https://github.com/Pdelam/Delamotte-et-al.-2024.git. For motility experiments, we monitored overnight tumor cell movements from *ex vivo* midgut tracts according to the long-live imaging technique.^89^ Ibidi dishes were placed overnight into a live-imaging chamber (25°C, 90% humidity, 0.1% CO2) and 20 image-mosaics were acquired with 5–7 Z-stacks every 20 min. We compared flybow tumorous cell movements using the TrackMate extension on Fiji.^90^

#### Immunostaining

For ACC immunostaining, digestive tracts were dissected in ice-cold PBS 1X buffer, placed in glass cups, fixed for 20 min at room temperature in a 4% paraformaldehyde (Electron Microscopy Sciences Catalog no.:15710) solution, rinsed for 10 min in 1X PBS, rinsed for 10 min in PBT (1X PBS, 0.1% Triton X-100) and saturated for 30 min in PBT with 2% bovine serum albumin; all at room temperature. Samples were incubated overnight at 4°C with the ACC primary antibody^43^ at 1:1000 in PBT. Samples were then rinsed three times in PBT for 10 min at room temperature before being incubated with a secondary antibody for two hours at room temperature. Tissues were rinsed in PBT for 10 min and incubated with DAPI at 1:500 for 30 min at room temperature. Finally, the samples were rinsed twice in 1X PBS and mounted in VECTASHIELD PLUS (ref. H-1900).

#### Other stainings

For neutral lipid staining, samples were dissected on ice and fixed as for immunostainings. After three 10-min washes in 1X PBS at room temperature, samples were incubated with LipidTox (ref. H34477) at 1:500 and DAPI at 1:500 at 4°C overnight. Subsequently, tissues were rinsed three times in 1X PBS and mounted in VECTASHIELD PLUS.

For mitochondria staining, samples were dissected and rinsed in 1X PBS buffer before being incubated in the staining solution [in 1X PBS, MitoTracker deep red (ref. M22426) at 1:10000, Hoechst 33342 at 2μg/mL] for 1 h at room temperature. Tissues were then rinsed two times in 1X PBS before being immediately mounted in 1X PBS and imaged.

### Quantification and statistical analysis

For cytometry, data consisted in the total cell count (average, 5474; SD, 2067) and the GFP cell count (average, 1379; SD, 1983) for 1778 samples (69 samples for the control, 29.0 ± 7.1 samples for 59 RNAi lines). Statistical differences with the control were assessed by a GLM using R version 4.1,^91^ considering that the proportion of GFP cells followed an overdispersed binomial distribution (“quasibinomial” family), the estimate of the dispersion parameter was 509.8. *p*-values were corrected for multiple testing using the method by.^92^ Effects of motility gene knockdowns were tested with the same statistical framework (quasibinomial GLM from GFP vs. all cells), from 102 samples (42 control). The dispersion parameter was 137, and *p*-values were also corrected for multiple testing.

For polyclonality, the flybow cassette was induced by heat-shocks of 30, 60, or 120 min, and the clone diversity was assessed at early (≤5-dpi) and long (≥20-dpi) times. Images were split into “tumor” and “clones” categories, “clones” serving as controls, for a total of 12 datasets. The recorded data consists in the number of images falling in 15 categories corresponding to all combinations of four colors: 4 categories with single colors (labeled G for “green”, R for “red”, Y for “yellow”, and B for “blue”), 6 categories with 2 colors (GR, GY, GB, RY, RB, and YB), 4 categories with 3 colors (GRY, GRB, GYB, RYB), and a single four-color category (GRYB). The data describing a combination of factors (for instance, tumors for 30-min heat-shock and long time) was thus a vector of size 15: **n**_30m,long,Tum_ = {n_G_, n_R_, …, n_GRYB_}.

The objective of the statistical treatment was to estimate the mean number of clones μ in each treatment, from the distribution of the observations **n**. This also required to estimate the frequency of induction of the four colors **p** = {p_G_, p_R_, p_Y_, p_B_}, which may vary according to the experimental conditions. Estimation of parameters **θ** = {μ, **p**} was performed by finding the values of these parameters that maximized the likelihood of observing **n**, assuming that the colors were induced independently in the different clones.

More precisely, the likelihood L(**θ** |**n**) = Prob(**n** |**θ**) = M(**n**, **F**(**θ**) ), where M stands for the multinomial probability of observing **n** counts in the 15 categories given the 15 theoretical frequencies:

$$F\;\left(\mu ,p\right)=\left[1/\left(1-P\left(0,\mu \right)\right)\right]\sum_{k>0}P\left(k,\mu \right)\;f\left(p,k\right)\text{,}$$

where P(k, μ) = μ^k^ e^−μ^/k! is the probability of observing k clones in an image when the average number of clones is μ, assuming a Poisson distribution. Note that frequencies had to be corrected by a factor 1/(1-P(0, μ)] since images without clones were discarded from the analysis. **f**(**p**, k) is the vector of probabilities for all color categories, which was determined by enumerating all combinations. For instance, f_G_(k) = p_G_^k^ (all k clones need to be green), f_GR_(k) = C_1,k-1_p_G_p_R_^k−1^ + C_2,k-2_p_G_^2^p_R_^k−2^ …, where C_a, b, c, d_ are multinomial coefficients.

In practice, the likelihood was maximized with the mle routine from the stats4 package in R v 4.1, 95% confidence intervals were estimated by profiling the likelihood function (confint.mle). Parameters were transformed for the fitting procedure (log transformation for μ, logistic transformation for the three first elements of **p**, the last one being deduced as p_B_ = 1 - p_G_ - p_R_ - p_Y_), and back-transformed to their original scale for the rest of the analysis.
