# Supplementary material for: Polyclonality and metabolic heterogeneity in a colorectal tumor model
Source: iScience. 2025 Jul 10;28(8):113090. doi: 10.1016/j.isci.2025.113090 (PMC12355558; doi:10.1016/j.isci.2025.113090)
Supplement: Document S1. Figures S1–S18, and Methods S1 [file mmc1.pdf]

iScience, Volume *28*

Supplemental information

Polyclonality and metabolic heterogeneity

in a colorectal tumor model

Pierre Delamotte, Mickael Poidevin, Yan Jaszczyszyn, Arnaud Le Rouzic, and Jacques Montagne

Sample name	Sample content	Final cell count
T_{ind1}	1*22-day old tumor	2273
T_{ind2}	1*22-day old tumor	1026
T_{ind3}	1*27-day old tumor	717
T_{ind4}	1*22-day old tumor	1097
T_{ind5}	1*21-day old tumor	903
T_{ind6}	1*21-day old tumor	493
T_{grp}	6*21-day old tumors	3405

Fig. S1: Samples used for single-cell RNAseq. The column sample content indicates the number of tumors included in each sample and their age. The number of cells recovered for RNAseq is indicated in the final column.

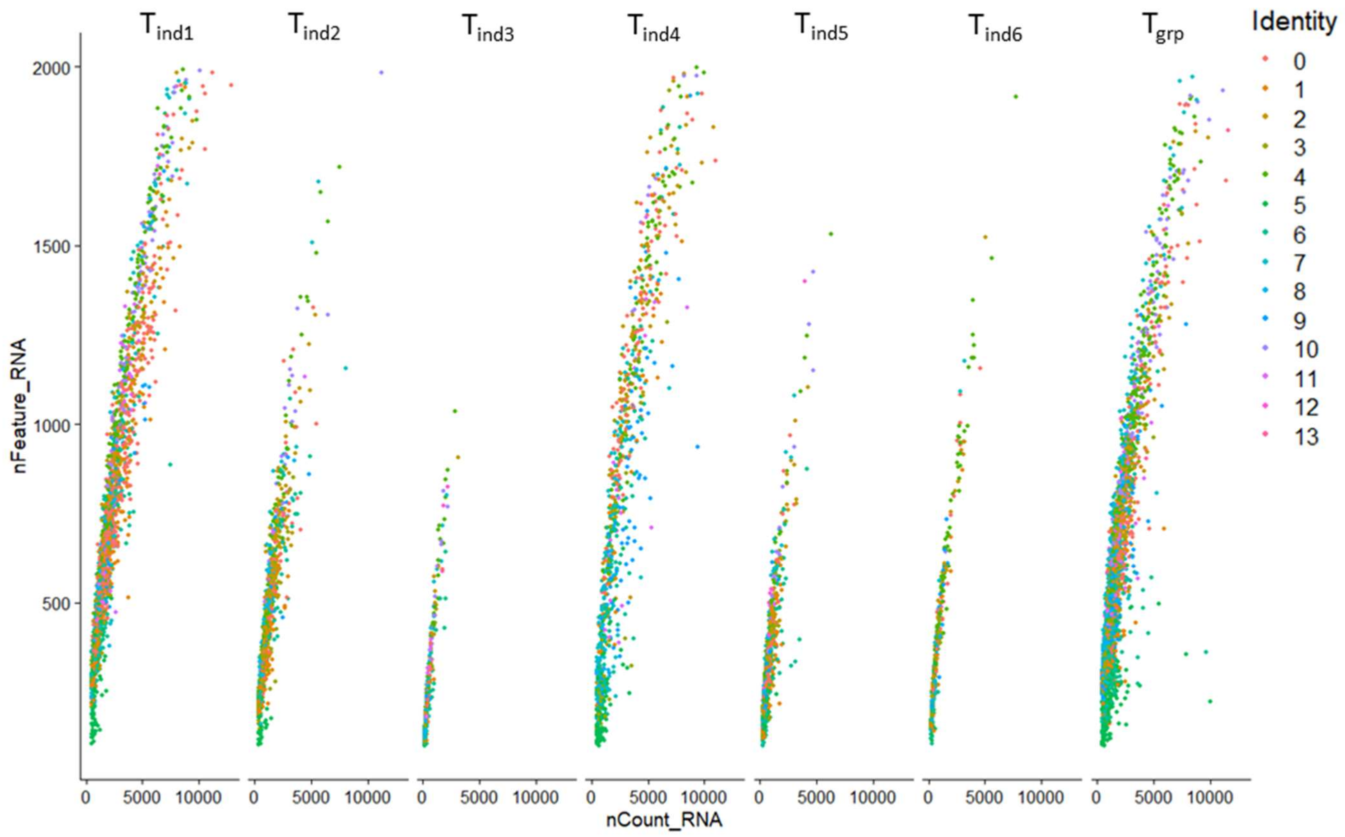

Fig. S2: Genomic cell signature of single-cell RNAseq samples. mRNA nFeature contents for each sample. The distributions do not reveal cluster bias.

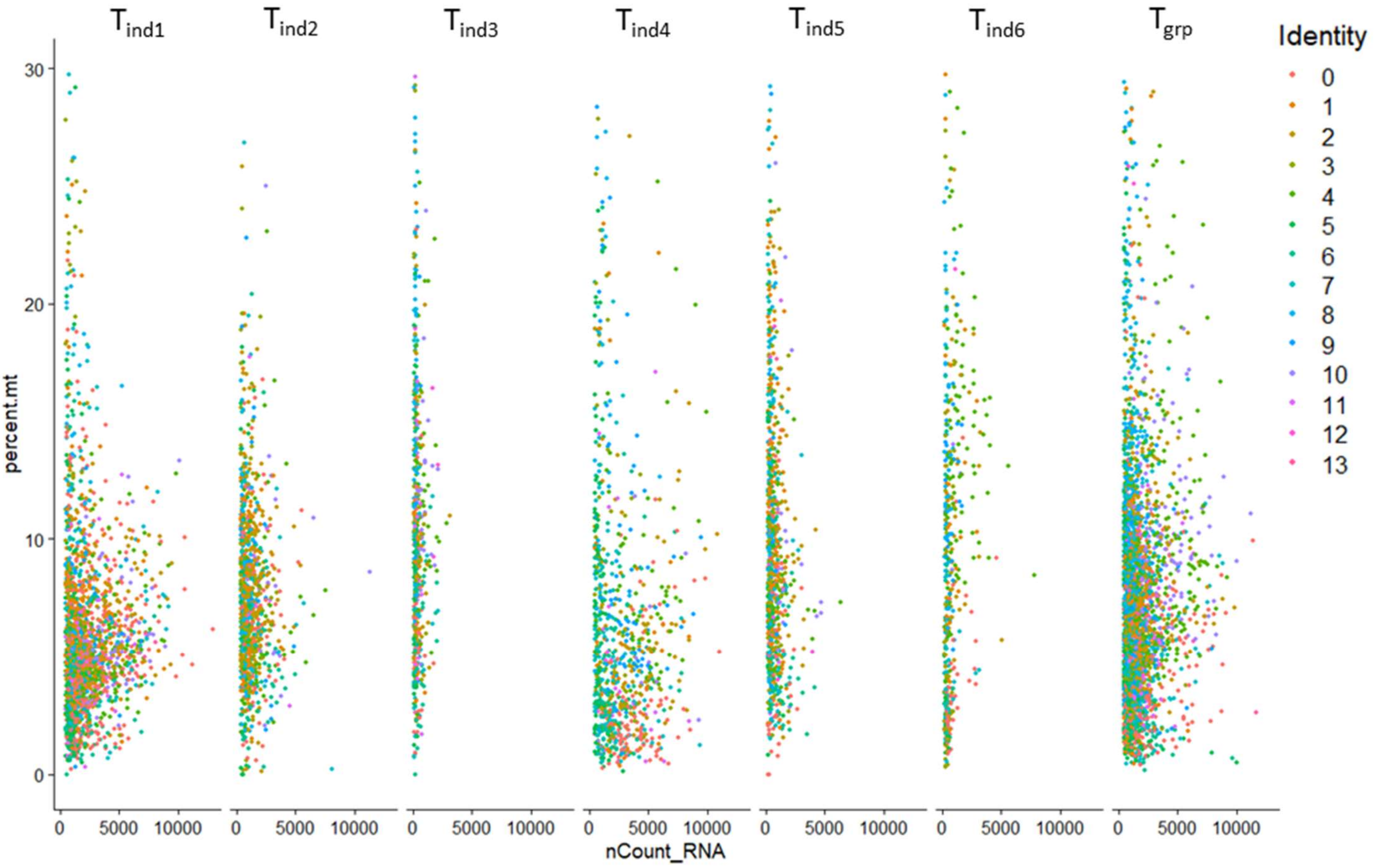

Fig. S3: Quality control of single-cell RNAseq samples. Mitochondrial RNA contents for each sample. Cells for which the value was above 15% were discarded. The distributions do not reveal a dead-cell cluster.

Cluster	T_{ind1}	T_{ind2}	T_{ind3}	T_{ind4}	T_{ind5}	T_{ind6}	T_{grp}
0	479	84	45	105	70	41	430
1	208	227	72	99	122	81	265
2	224	156	46	64	72	37	357
3	224	107	64	118	95	31	242
4	109	46	49	107	57	38	356
5	193	125	24	41	92	37	155
6	124	41	34	75	36	5	289
7	63	27	18	86	20	24	322
8	98	28	35	21	52	52	199
9	131	47	21	50	31	33	154
10	123	52	38	24	33	4	176
11	151	25	27	38	14	5	135
12	0	2	6	0	7	1	30
13	5	0	0	0	0	0	11

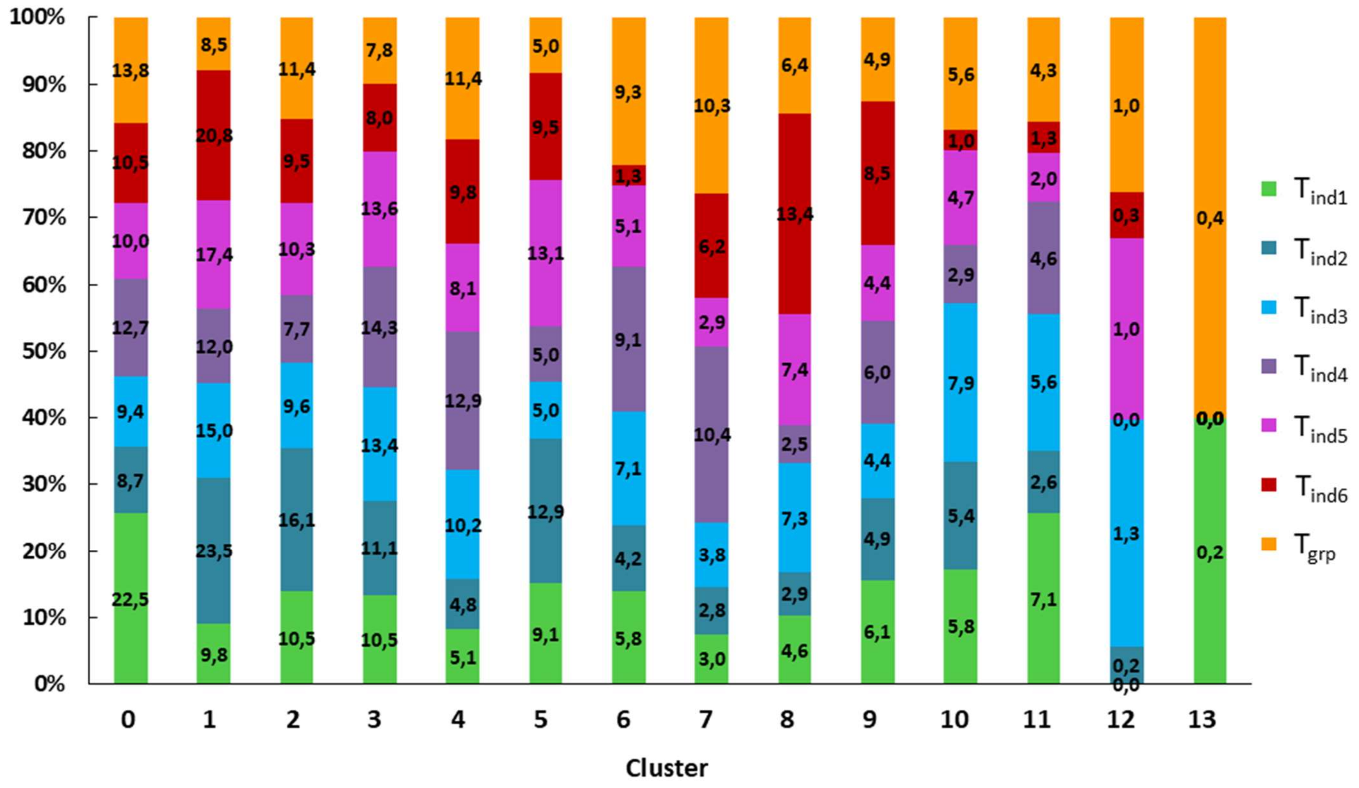

Fig. S4: Tumor cell distribution within clusters. Cell counts in each cluster for each sample (top) and the contribution of each sample for each cluster showing that all individual tumors contain at least clusters 0 to 11 (bottom).

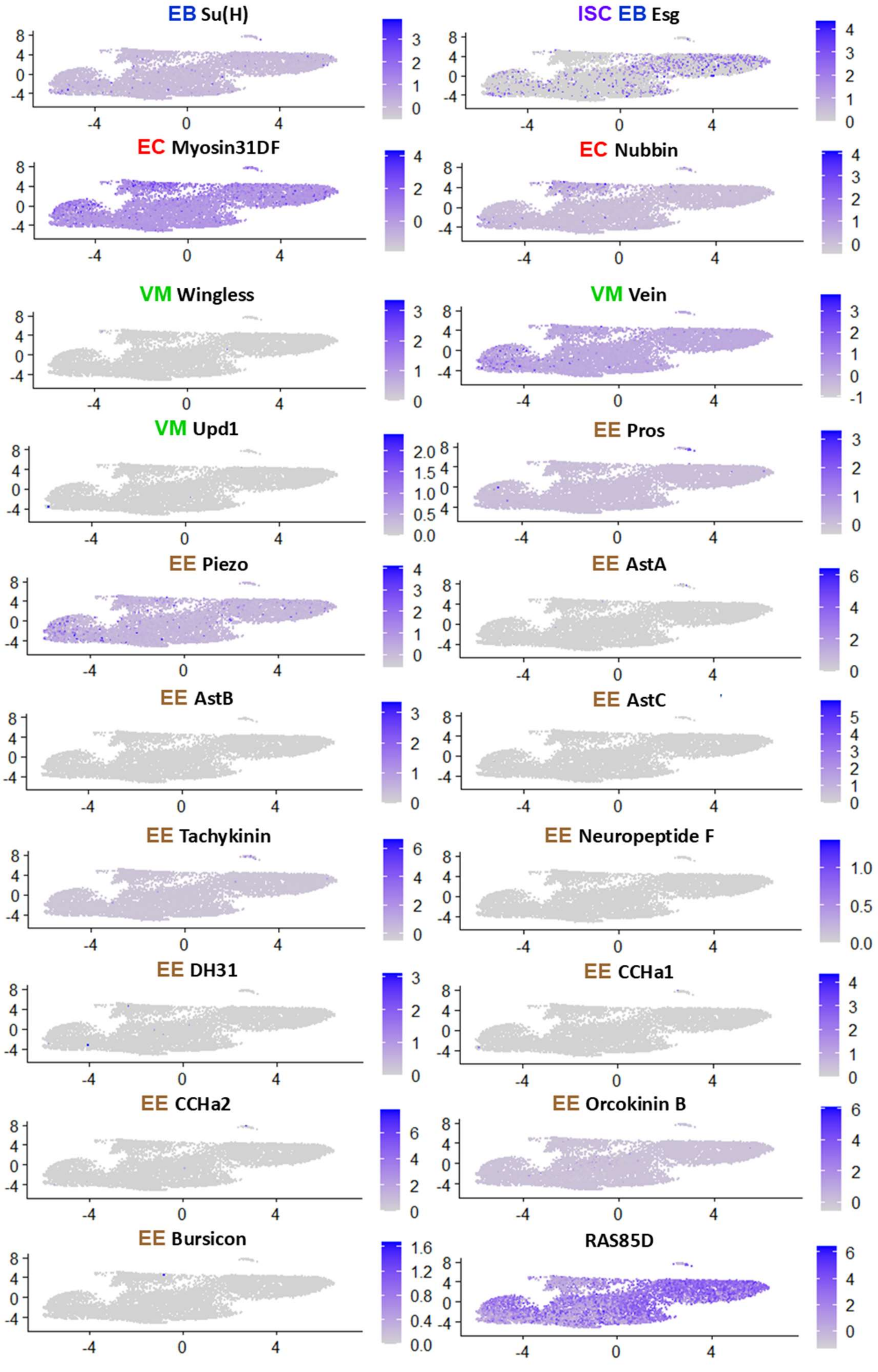

Fig. S5: Intestinal cell markers. Feature-plots from single-cell RNAseq pool based on intestinal cell markers [1]: intestinal stem cell (ISC), enteroblast (EB), enterocyte (EC), enteroendocrine (EE) and visceral muscles (VM).

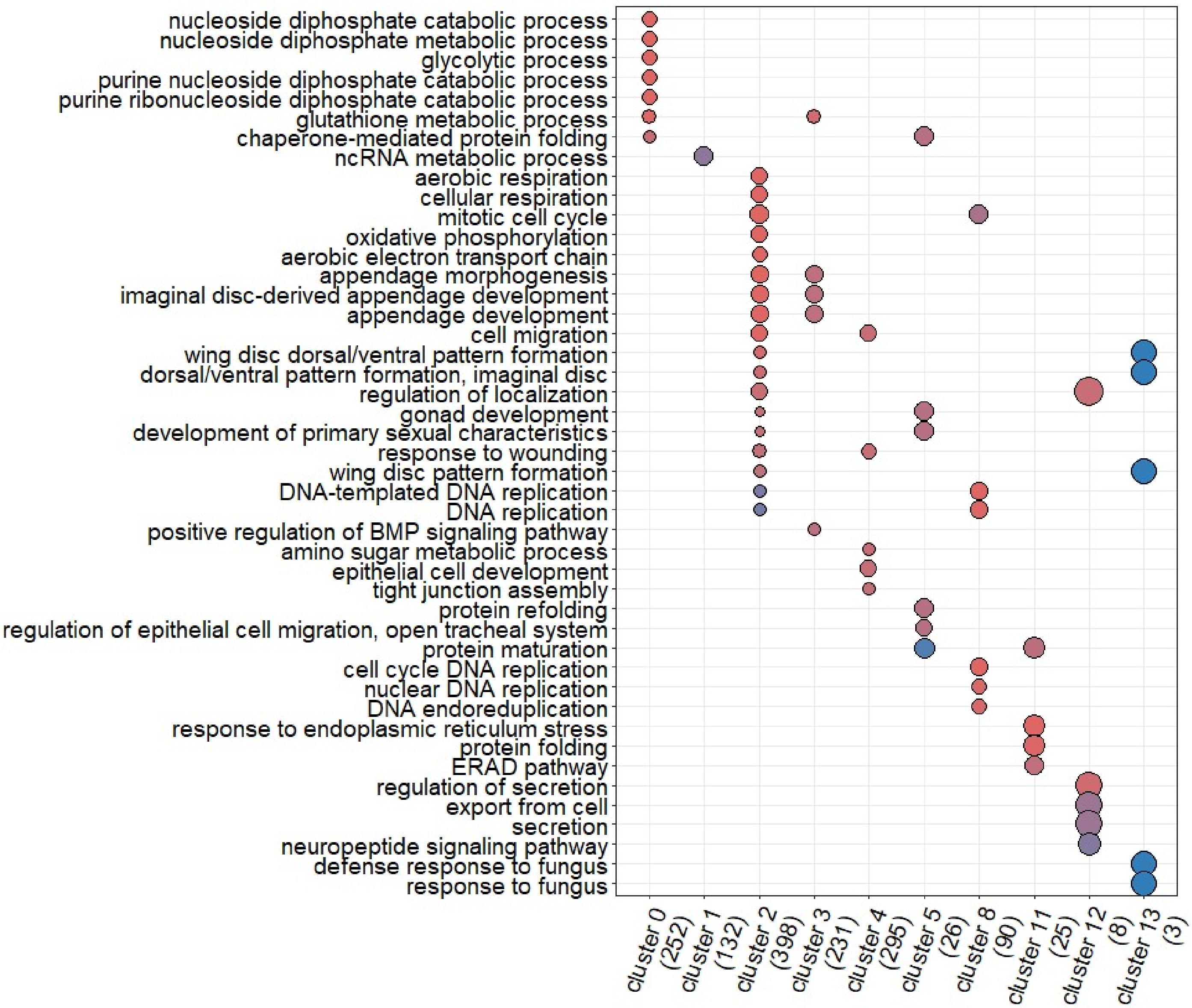

Fig. S6: Gene ontology. Analysis from single-cell RNAseq pool for biological processes.

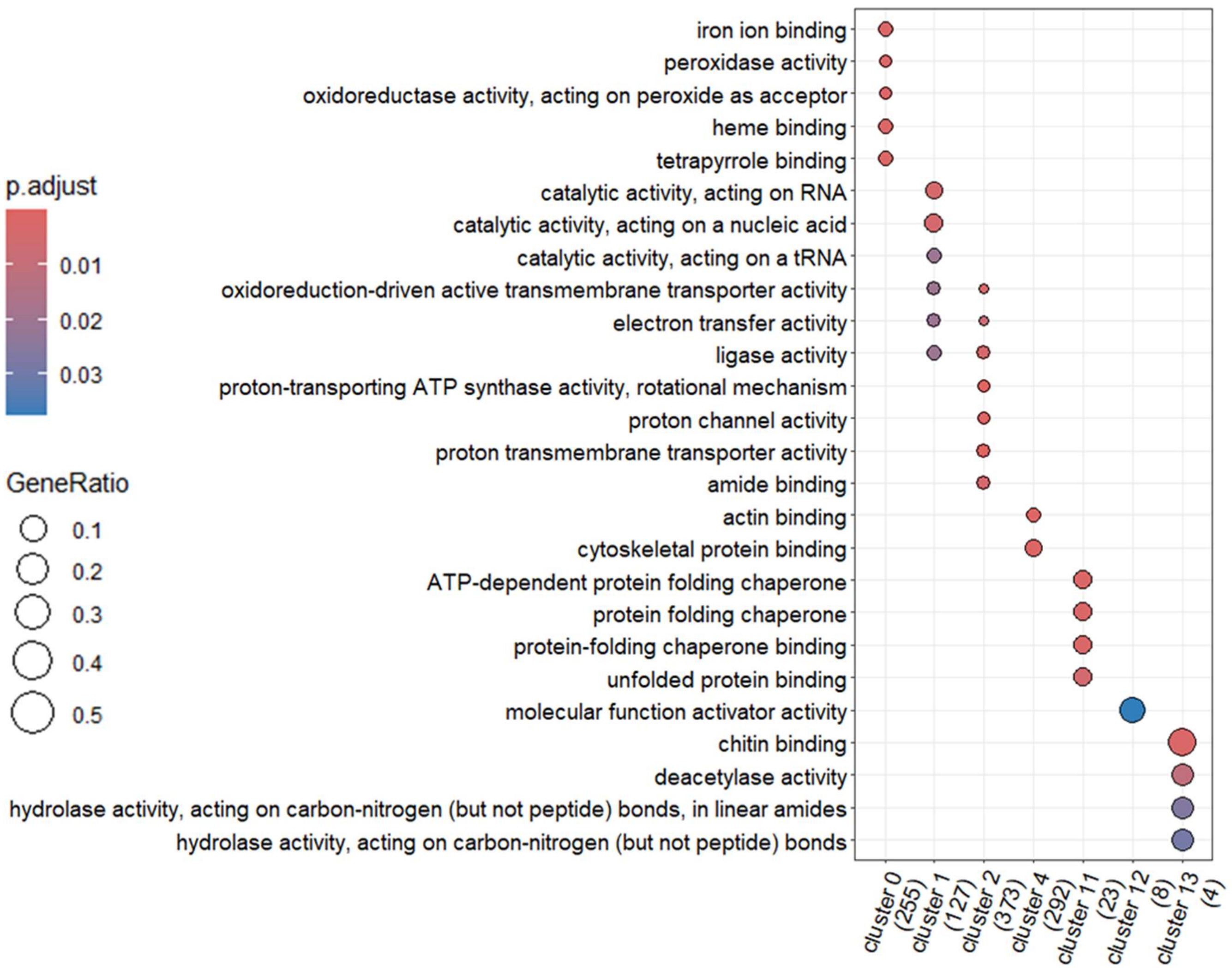

Fig. S7: Gene ontology. Analysis from single-cell RNAseq pool for molecular functions.

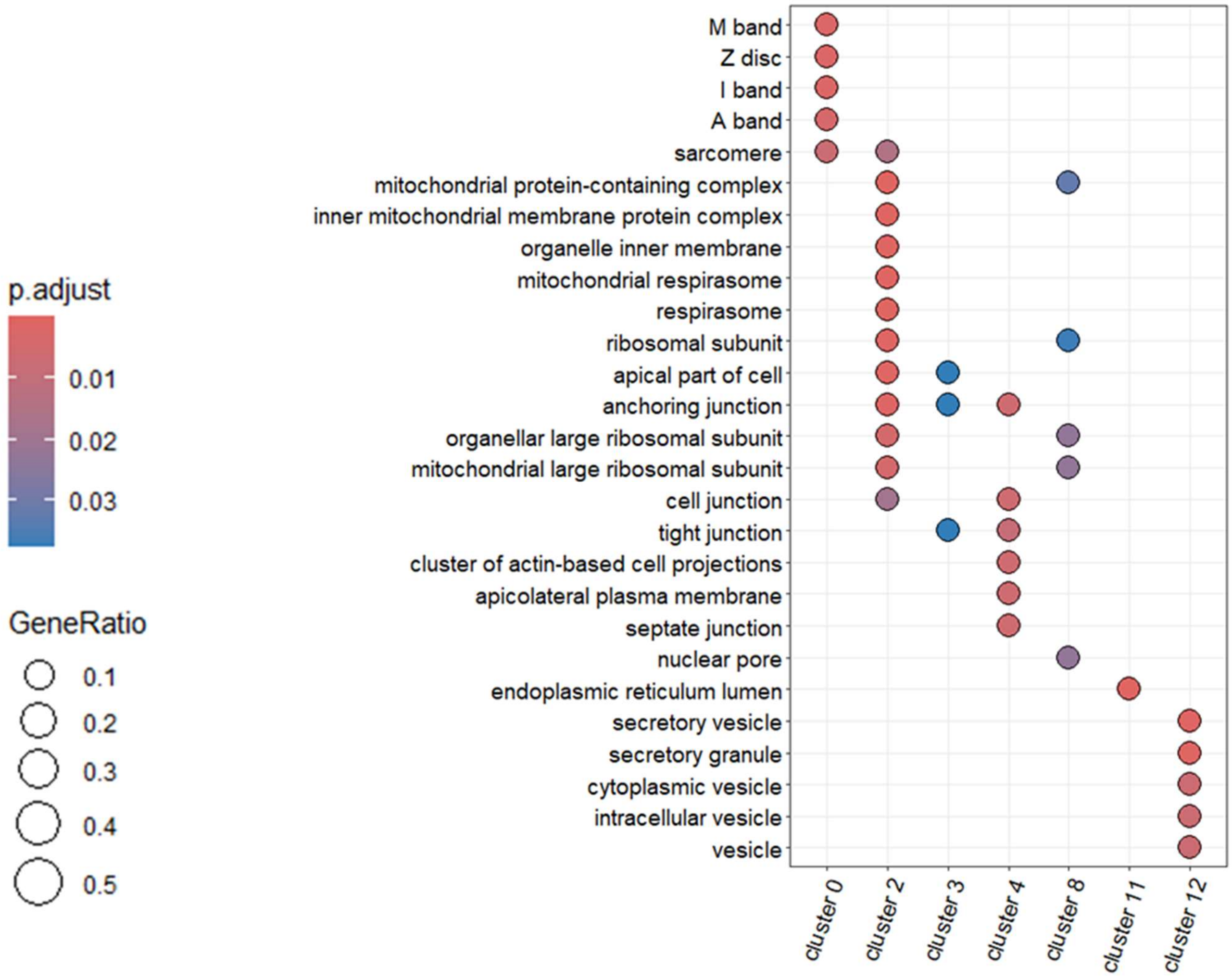

Fig. S8: Gene ontology. Analysis from single-cell RNAseq pool based on the five best hits for each cluster.

Cluster	ID	Description	GeneRatio
cluster 0	GO:0019752	carboxylic acid metabolic process	21/252
cluster 0	GO:0043436	oxoacid metabolic process	21/252
cluster 0	GO:0005975	carbohydrate metabolic process	19/252
cluster 0	GO:0044255	cellular lipid metabolic process	18/252
cluster 0	GO:0032787	monocarboxylic acid metabolic process	18/252
cluster 0	GO:0006753	nucleoside phosphate metabolic process	17/252
cluster 0	GO:0009117	nucleotide metabolic process	16/252
cluster 0	GO:0044270	cellular nitrogen compound catabolic process	14/252
cluster 0	GO:1901136	carbohydrate derivative catabolic process	13/252
cluster 0	GO:0005996	monosaccharide metabolic process	13/252
cluster 2	GO:0048870	cell motility	30/398
cluster 2	GO:0016477	cell migration	28/398
cluster 2	GO:0015980	energy derivation by oxidation of organic compounds	28/398
cluster 2	GO:0009060	aerobic respiration	27/398
cluster 2	GO:0045333	cellular respiration	27/398
cluster 2	GO:0030036	actin cytoskeleton organization	25/398
cluster 2	GO:0030029	actin filament-based process	25/398
cluster 2	GO:0006753	nucleoside phosphate metabolic process	25/398
cluster 2	GO:0009117	nucleotide metabolic process	25/398
cluster 2	GO:0006119	oxidative phosphorylation	23/398
cluster 3	GO:0006575	cellular modified amino acid metabolic process	9/231
cluster 3	GO:0006749	glutathione metabolic process	8/231
cluster 4	GO:0016477	cell migration	22/295
cluster 4	GO:0048870	cell motility	22/295
cluster 4	GO:0044255	cellular lipid metabolic process	22/295
cluster 4	GO:0007015	actin filament organization	13/295
cluster 4	GO:0006040	amino sugar metabolic process	7/295
cluster 4	GO:0006022	aminoglycan metabolic process	7/295
cluster 4	GO:0045010	actin nucleation	5/295
cluster 4	GO:1901071	glucosamine-containing compound metabolic process	5/295
cluster 4	GO:0034314	Arp2/3 complex-mediated actin nucleation	4/295
cluster 5	GO:2000274	regulation of epithelial cell migration, open tracheal system	2/26

Fig. S9: Gene ontology. Detail of 10 best metabolic and motility hits from biological processes.

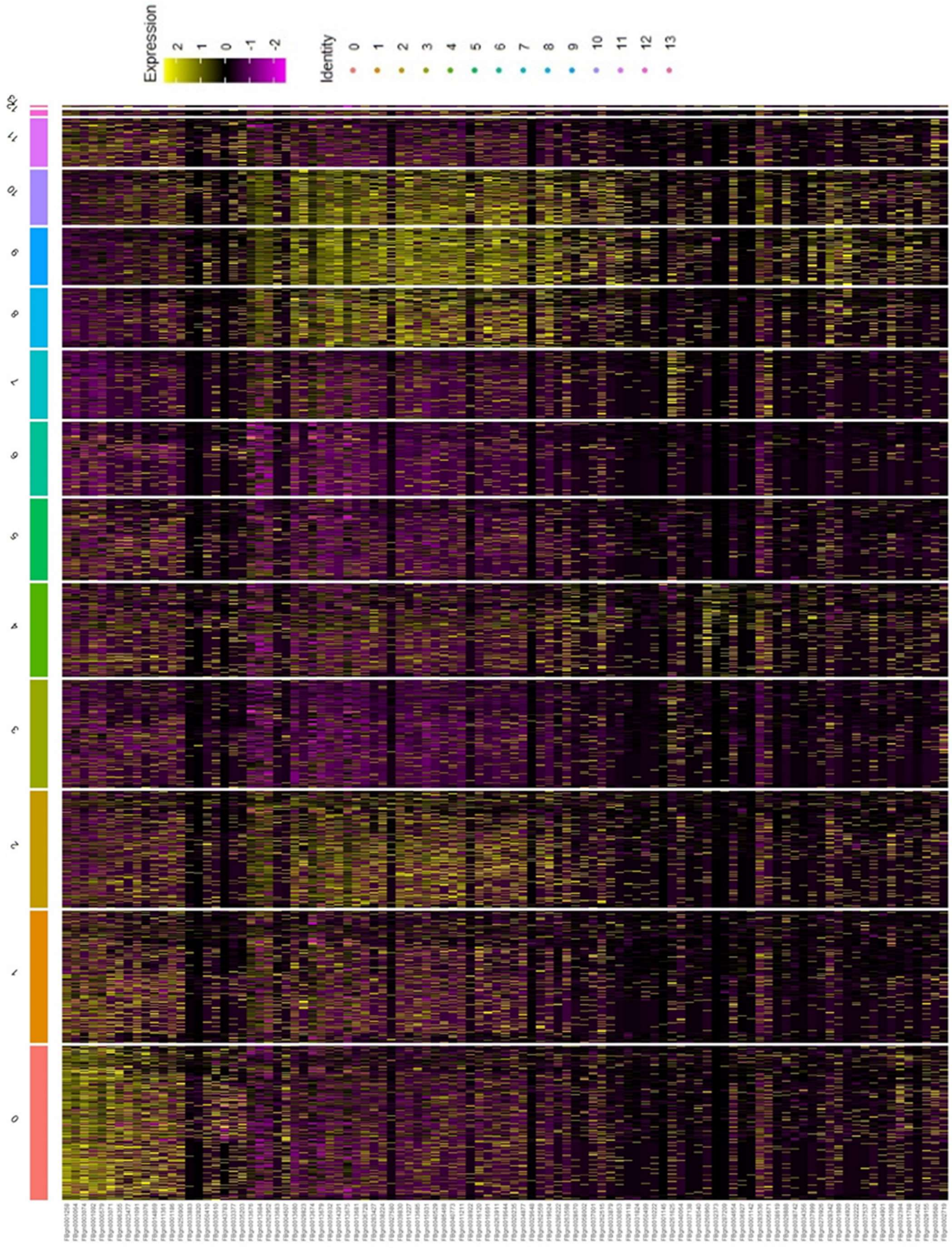

Fig. S10. Metabolic heatmap. The heatmap has been generated from single-cell RNAseq pool based on metabolic KEGG pathway lists.

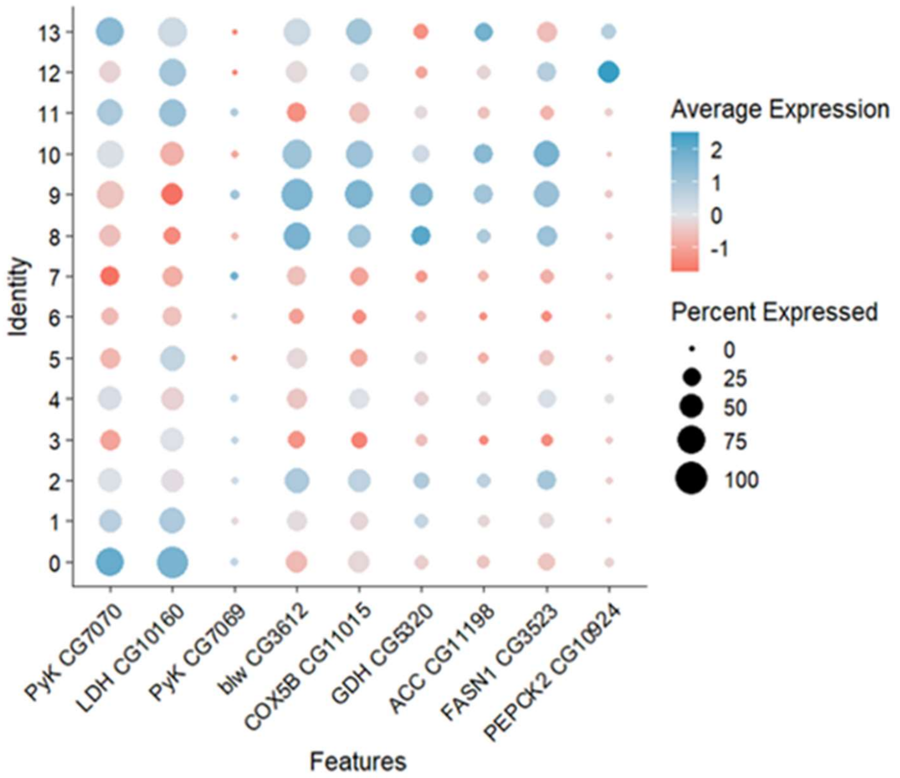

Fig. S11: Cluster segregation of a few metabolic genes. A selection of metabolic genes screened for their requirement in tumor growth (Fig. 2) exhibits cluster-specific segregation**.**

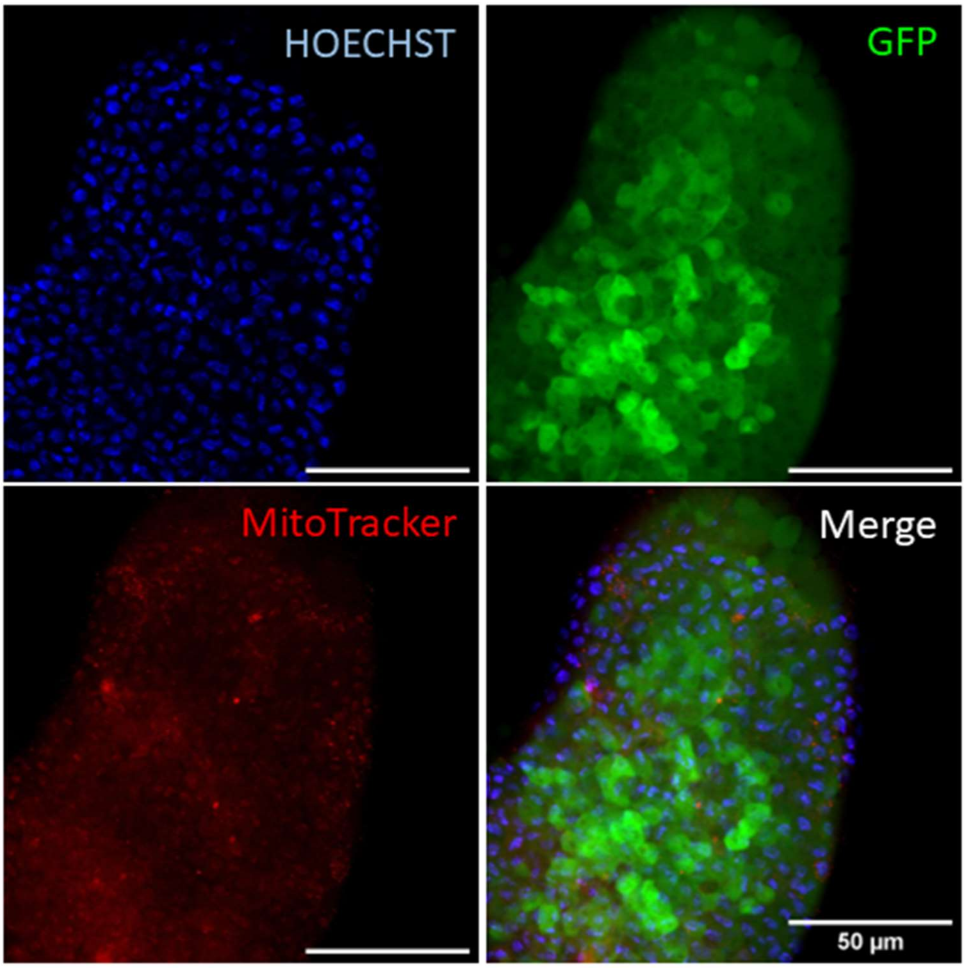

Fig. S12. Mitochondrial staining heterogeneity in tumors. Nuclear staining (HOECHST), GFP-labelled tumor (GFP), mitochondria (MitoTracker) and merged image (Merge).

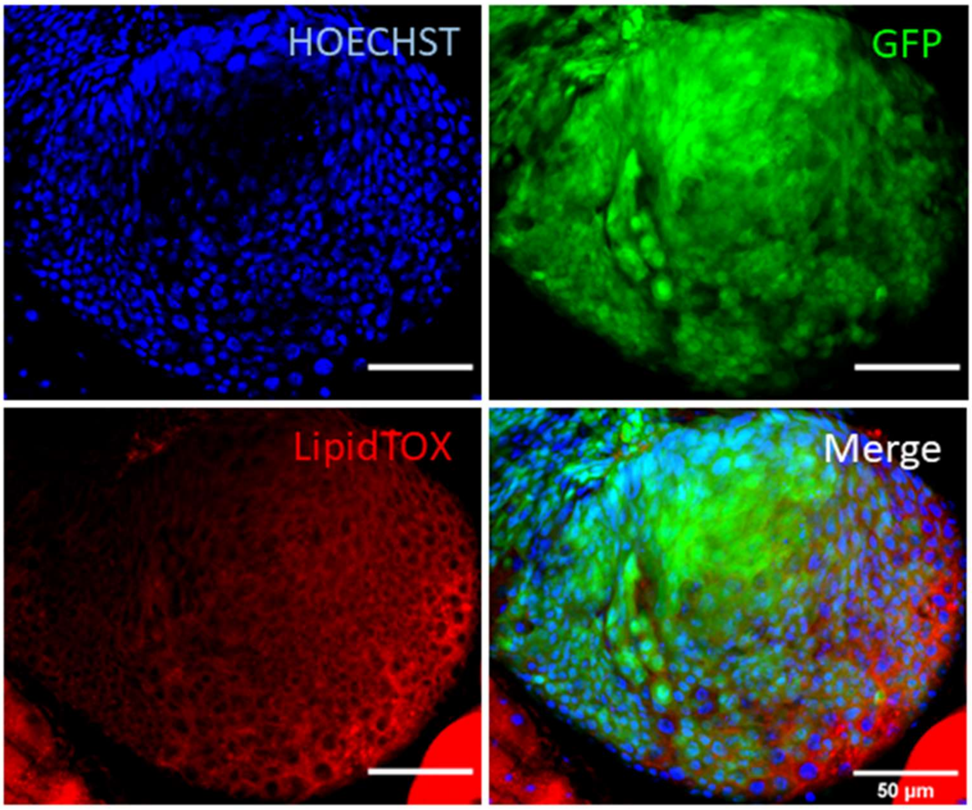

Fig. S13. Lipid content heterogeneity in tumors. Nuclear staining (HOECHST), GFP-labelled tumor (GFP), lipids (LipidTOX) and merged image (Merge).

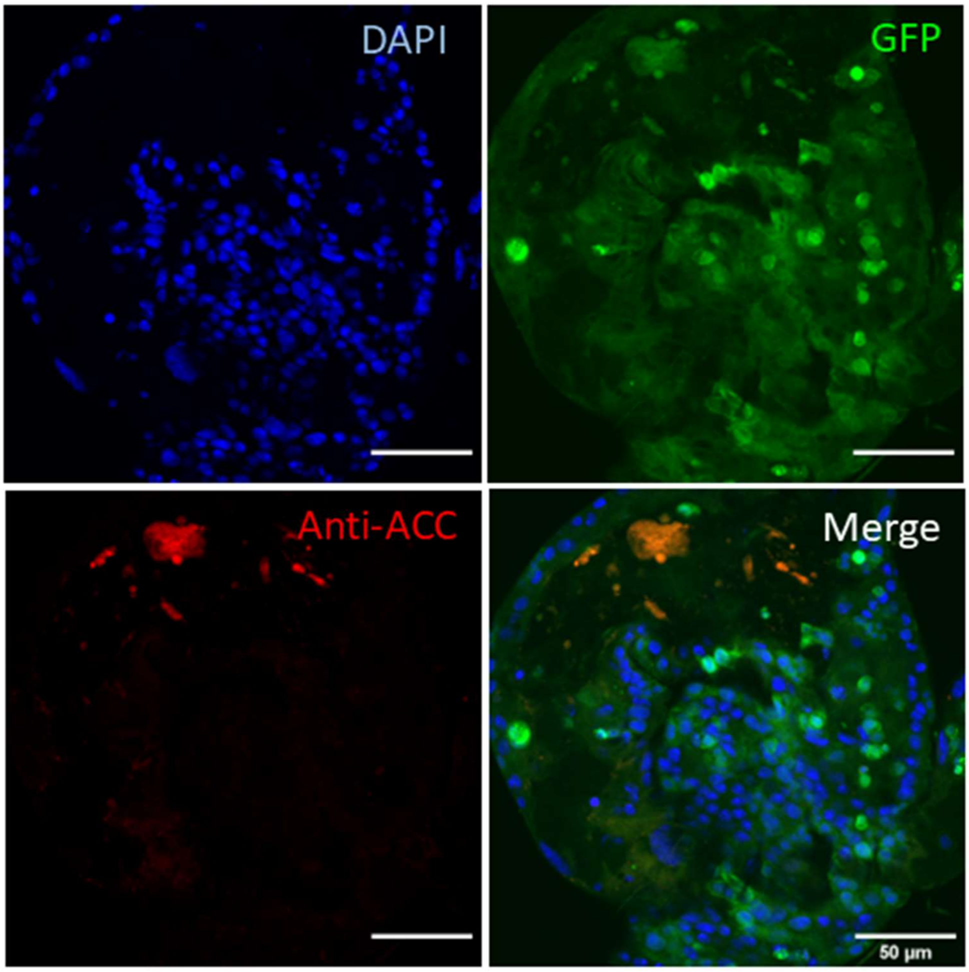

Fig. S14. ACC heterogeneity in tumors. Nuclear staining (DAPI), GFP-labelled tumor (GFP), ACC antibody staining (Anti-ACC) and merged image (Merge).

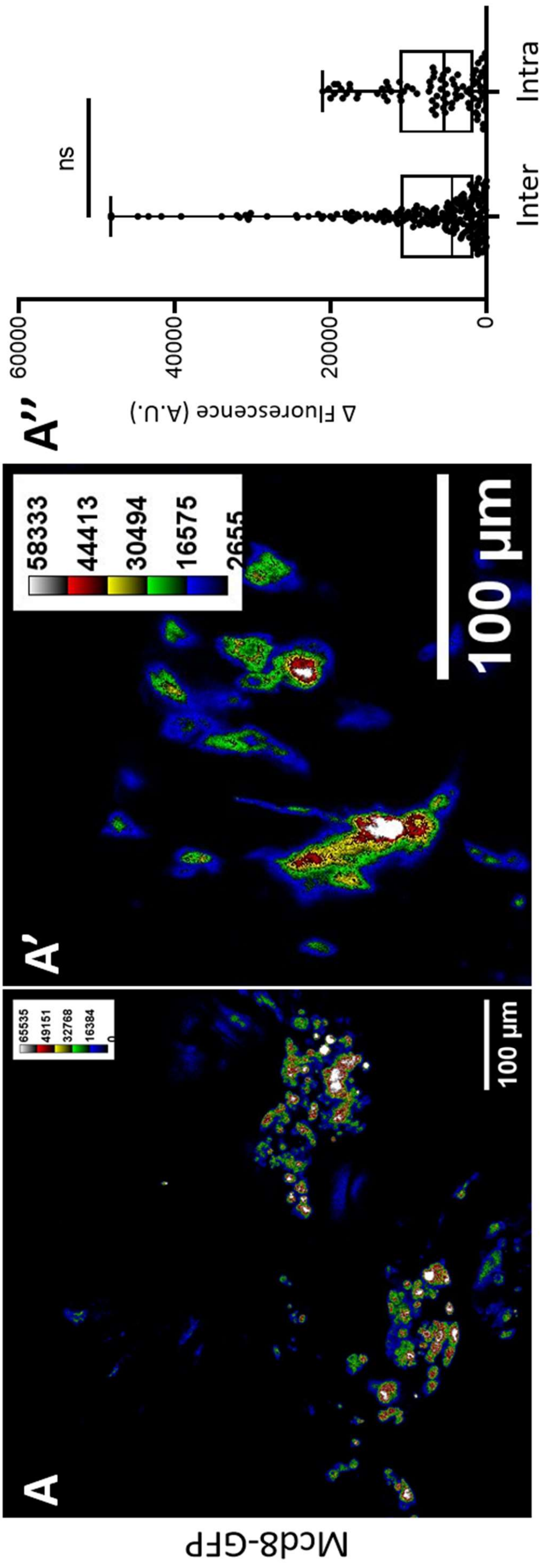

Fig. S15. GFP expression in tumors and clones. (**A-A'**) GFP in tumors (**A**) and clones (**A'**) shows variable expression levels. (**A''**) Fluorescence quantification between cells of the same clone (intra) to cells from distant clones (inter), shows no significant difference.

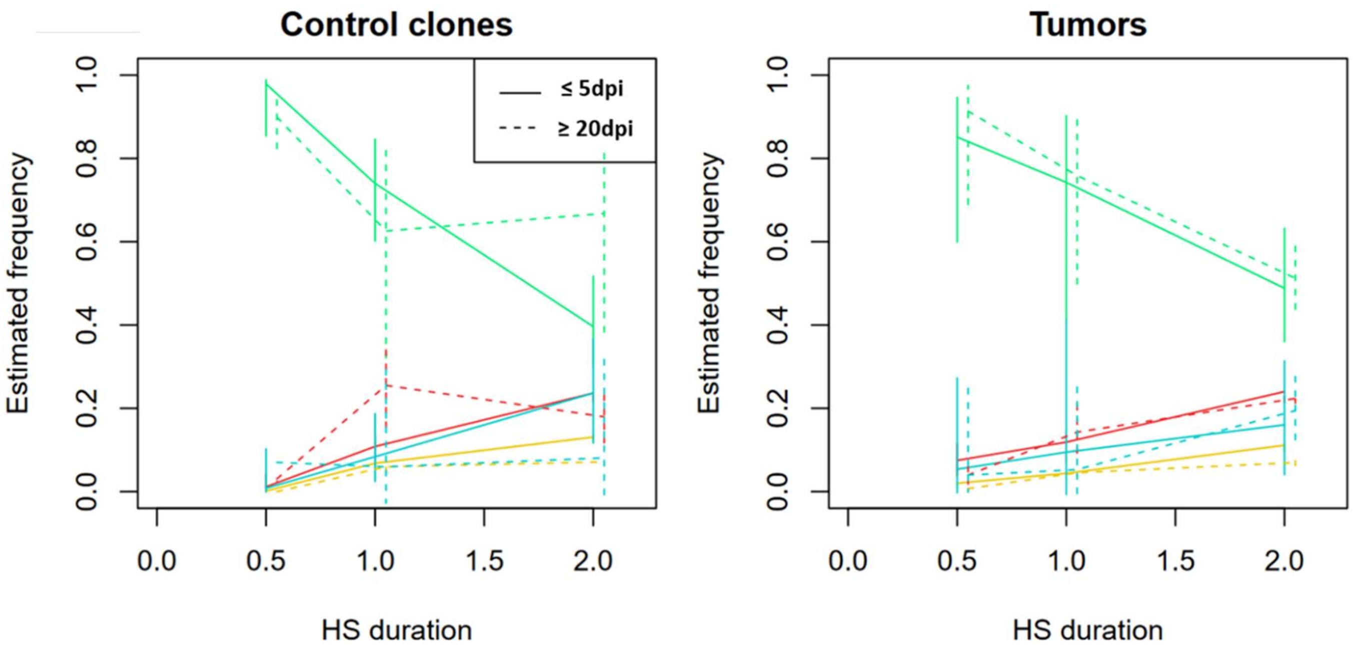

Fig. S16: Fluorophore distribution in clones and tumors. Estimated fluorophore occurrence in control clones (left) and tumors (right), based on the presence of each fluorophore in the image dataset. GFP (green), mCherry (red), mTurquoise2 (cyan) and mCitrine (yellow) occurrences were modelized at for 30-, 60- and 120-mins induction times (x-axis) and observed at early (plain line) and late (dotted line) stages.

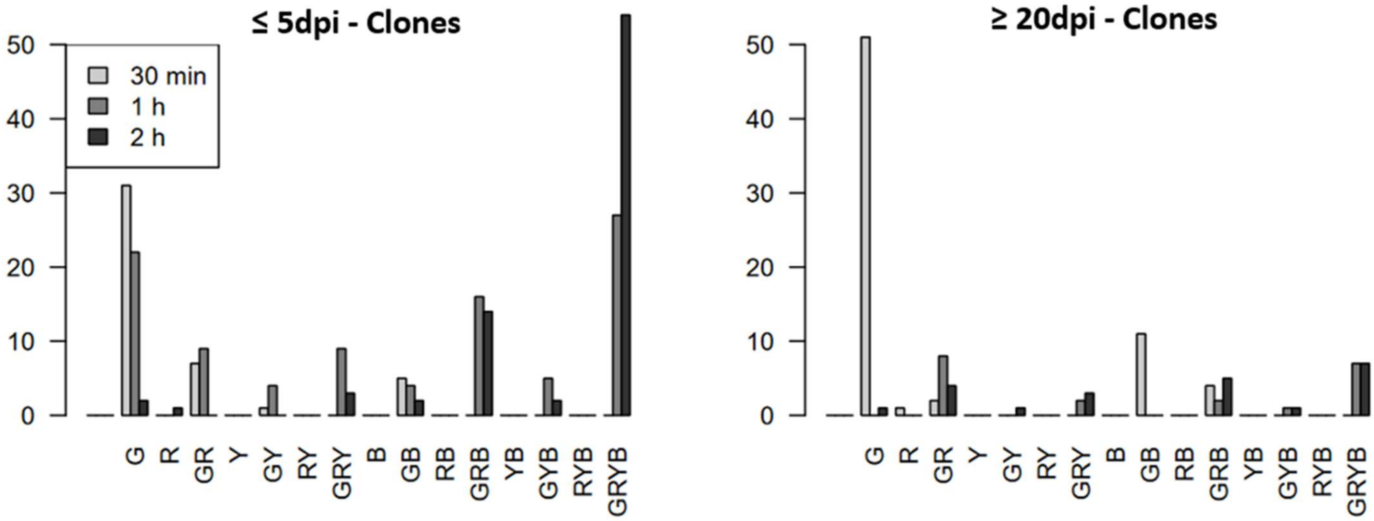

Fig. S17: Fluorophore distribution in clones. Fluorophore distribution seen in the image dataset at early (left) and late (right) stages. Dataset for clones recorded the presence or absence of each fluorophore in all clones seen in the field of each image. The y-axes indicate the number of images seen for each combination.

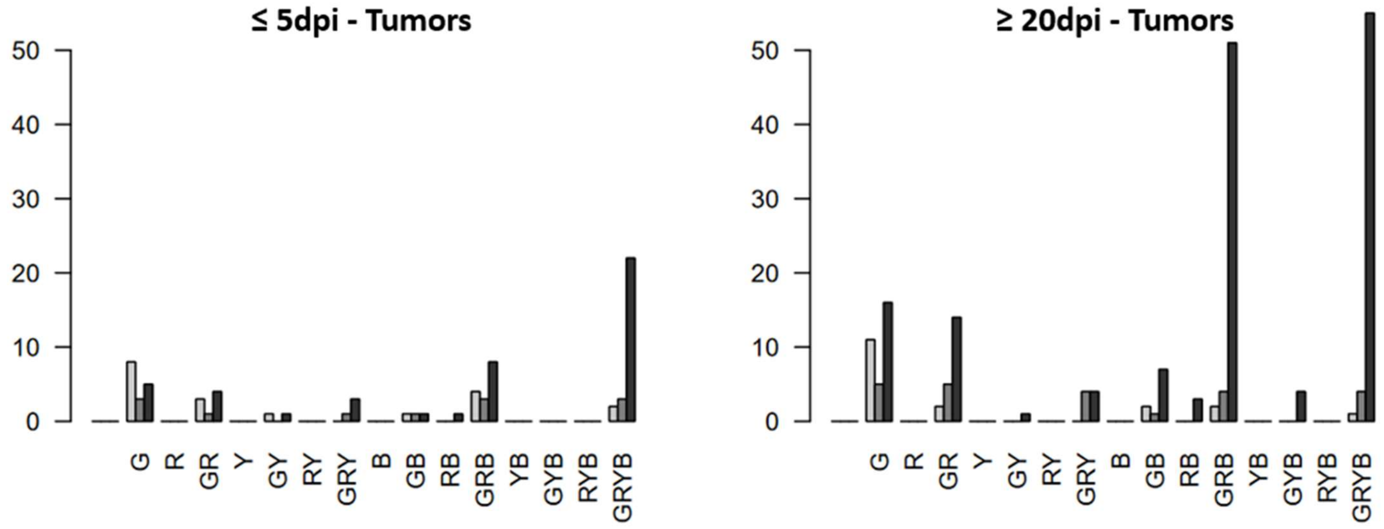

Fig. S18: Fluorophore distribution in tumors. Fluorophore distribution seen in the image dataset at early (left) and late (right) stages. Dataset for tumors recorded the presence or absence of each fluorophore in the delineated tumors of each image. The y-axes indicate the number of images seen for each combination.

Methods S1: Physiological and genetic information for the *Drosophila* model

The *Drosophila* digestive tract:

The digestive tract of insects comprises the foregut and the hindgut of ectodermal origin and the midgut of endodermal origin. As for most insect species, the midgut is the central digestive region of *Drosophila* [1]. The midgut is a monolayer epithelium that contains i) enterocytes (ECs), also called columnar cells, ii) enteroendocrine cells (EECs), and iii) intestinal stem cells (ISCs). ISCs and EECs are diploid cells, whereas ECs are polytenic after a set of endoreplication. The division of ISC generates a new ISC and either an EEC precursor or an enteroblast (EB) that will differentiate into an EEC or an enterocyte, respectively. Each cell type and precursor is characterized by a specific gene expression set. For instance, the gene *escargot* (*esg*) is expressed in ISCs and EBs but not in differentiated cells [2]. In this study, we used the bipartite gal4>UAS system to express GFP, RasV12 and a specific interfering RNA (RNAi) in *esg-positive* cells. This system includes a gal4 transgenic line where the *esg* promoter directs the expression of the yeast transcription factor Gal4 and UAS transgenic lines where the UAS sequences responsive to Gal4 direct the expression of GFP, RasV12 or RNAi. After crossing *esg-gal4* with UAS transgenic flies, their progeny will express RASV12 in combination with GFP, RNAi or a metabolic sensor in ISCs and EBs.

The genetic strategy to generate somatic clones:

The *Drosophila* genome comprises four chromosomes (X, II, III and IV). The X being the gonosome, the II, III and IV the autosomes, with the latter being very small for which genetics is limited. In our study, mutations and transgenes reside on chromosomes X, II and III. The X chromosome bears a heat-shock induced flipase (flp) transgene and in combination with a heat-shock induced specific flipase (flp5) when using the flybow transgene [3]. One of the parental line bears on chromosome II the *esg*-gal4 transgene in combination with either a UAS-gal4 or the flybow transgene, and on chromosome III a recombination cassette that allows an flp-induced exchange of the right arm of chromosome III, which also contains a transgene ubiquitously expressing the Gal4-inhibitor Gal80. The other parental line bears on the chromosome II the UAS-RasV12 transgene in combination with either a UAS-RNAi or a UAS-metabolic sensor, and on the right arm of chromosome III the flp-induced exchange cassette and the adenomatous polyposis coli (*Apc*) mutations; see also [4,5]. After crossing these two parental lines, their progeny will develop tumors after heat-shock induced somatic recombination [6]. Heat-shock induced flp may produce exchange of the right arm of chromosome III, so that after cell division, some daughter cells may become homozygous for the *Apc* mutations, lacking the ubiquitous Gal80 (Scheme below).

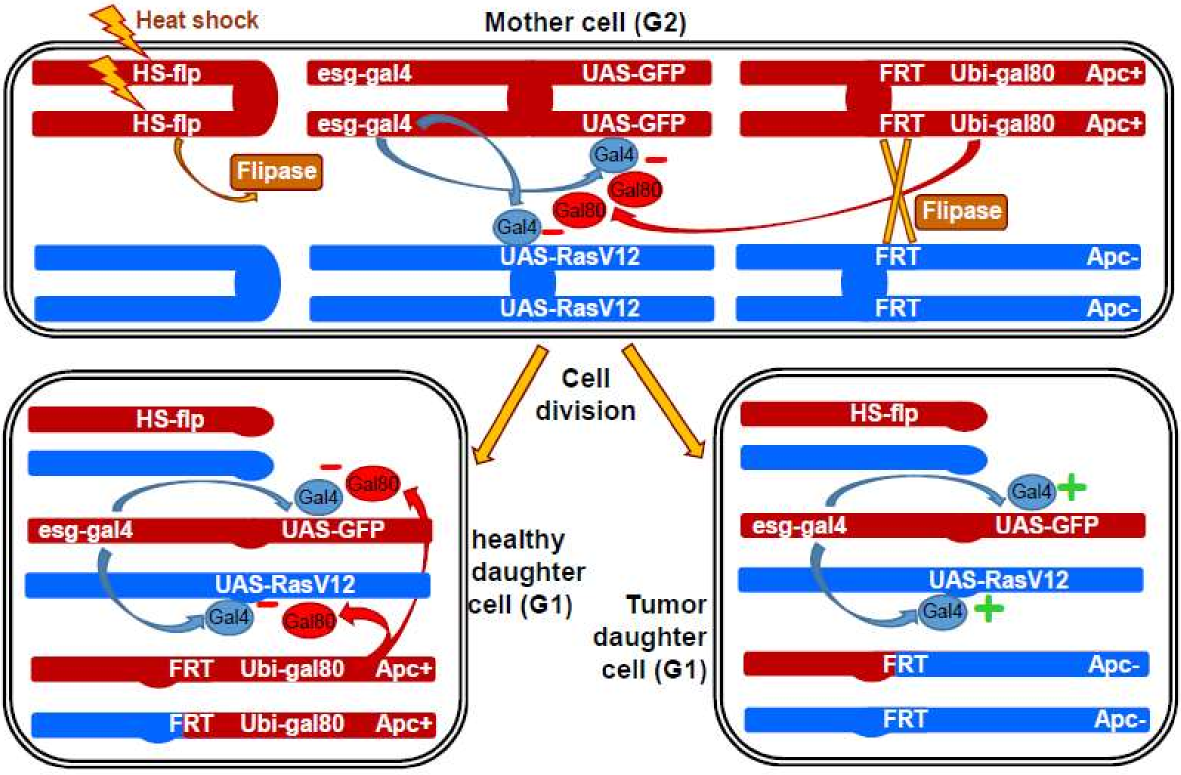

***Genetic scheme for generating tumor clones in the midgut**: The mother cell before division is in the G2 stage. The flipase induced by heat shock may produce exchange of the right arms of chromosome III that contains a FRT sequence. One of the possible arm exchange may result in the formation of tumor cell as depicted here.*

Subsequently, Gal4 becomes active only in these *Apc* homozygous cells to express RasV12 and any of the other UAS transgenes. The other daughter cells being homozygous wild-type for Apc and ubiquitously expressing Gal80 is not tumoral and do not express GFP. For the flybow approach, the heat-shock also induces flp5 that permits reorganization of the flybow transgene and expression of either of the four fluorophores [3], although not with an equal frequency, the GFP fluorophore being the most prominent one. Therefore, given that our predictive model revealed that tumors must derive from about eight founder clones, a tumor must contain a few clones labelled by the same fluorophore. Consistent with the highest frequency of the GFP fluorophore, we seldom observed mono-labelled tumors by GFP, but never by any of the three other fluorophores, which further supports the obligate polyclonality.

Intestinal tumors in *Drosophila*:

Other intestinal tumor models have been established in *Drosophila*; the interest of the one we used relies on *Apc* loss-of-function and ectopic expression of an oncogenic form of Ras, two events frequently reported in human CRC [7]. In this strategy, only a few cells of the midgut are properly recombined to produce tumors, which sometimes bud from the intestinal epithelium [4]. Our tumor model may generate several recombined cells, each being a potential founder clones, which do not necessarily reflects the putative frequency of CRC precursors in Human. However, old *Drosophila* flies develop spontaneous intestinal tumors at very low frequency, likely because of their short lifespan [8]. Whether these spontaneous tumors are also polyclonal should be investigated in the future.

Supplemental references:

1. Delamotte, P., and Montagne, J. (2024). Dietary Lipids and Their Metabolism in the Midgut. Adv Exp Med Biol. 10.1007/5584_2024_835.
2. Toledano, H., D'Alterio, C., Loza-Coll, M., and Jones, D.L. (2012). Dual fluorescence detection of protein and RNA in Drosophila tissues. Nat Protoc *7*, 1808-1817. 10.1038/nprot.2012.105.
3. Hadjieconomou, D., Rotkopf, S., Alexandre, C., Bell, D.M., Dickson, B.J., and Salecker, I. (2011). Flybow: genetic multicolor cell labeling for neural circuit analysis in Drosophila melanogaster. Nat Methods *8*, 260-266. 10.1038/nmeth.1567.
4. Martorell, O., Merlos-Suarez, A., Campbell, K., Barriga, F.M., Christov, C.P., Miguel-Aliaga, I., Batlle, E., Casanova, J., and Casali, A. (2014). Conserved mechanisms of tumorigenesis in the Drosophila adult midgut. PLoS One *9*, e88413. 10.1371/journal.pone.0088413
5. Dawson, E.H., Bailly, T.P.M., Dos Santos, J., Moreno, C., Devilliers, M., Maroni, B., Sueur, C., Casali, A., Ujvari, B., Thomas, F., et al. (2018). Social environment mediates cancer progression in Drosophila. Nat Commun *9*, 3574. 10.1038/s41467-018-05737-w.
6. Lee, T., and Luo, L. (2001). Mosaic analysis with a repressible cell marker (MARCM) for Drosophila neural development. Trends Neurosci *24*, 251-254.
7. Smith, G., Carey, F.A., Beattie, J., Wilkie, M.J., Lightfoot, T.J., Coxhead, J., Garner, R.C., Steele, R.J., and Wolf, C.R. (2002). Mutations in APC, Kirsten-ras, and p53--alternative genetic pathways to colorectal cancer. Proc Natl Acad Sci U S A *99*, 9433-9438. 10.1073/pnas.122612899.
8. Salomon, R.N., and Jackson, F.R. (2008). Tumors of testis and midgut in aging flies. Fly (Austin) *2*, 265-268. 10.4161/fly.7396.
